# Assessing dissociation: A systematic review and evaluation of existing measures

**DOI:** 10.1016/j.jpsychires.2024.11.040

**Published:** 2025-01

**Authors:** Sorawit Wainipitapong, L.S. Merritt Millman, Xi Huang, Lillian Wieder, Devin B. Terhune, Susannah Pick

**Affiliations:** a Department of Global Health and Social Medicine, King's College London, London, United Kingdom; b Department of Psychiatry and Center of Excellence in Transgender Health, Faculty of Medicine, Chulalongkorn University and King Chulalongkorn Memorial Hospital, Bangkok, Thailand; c Department of Psychological Medicine, Institute of Psychiatry, Psychology and Neuroscience, King's College London, London, United Kingdom; d Department of Psychology, Institute of Psychiatry, Psychology, and Neuroscience, King's College London, London, United Kingdom

**Keywords:** Dissociation, Measure, Tool, Questionnaire, Psychometric properties, COSMIN

## Abstract

**Objective:**

This review aimed to assess the psychometric properties and methodological quality of existing dissociation measures.

**Methods:**

MEDLINE, Embase, and PsycINFO were searched in May 2023 using comprehensive search terms for ‘dissociation’ combined with terms for ‘measurement’ and ‘psychometric properties’. The review was registered with PROSPERO (CRD42023423485) and followed PRISMA and COSMIN guidelines. We assessed content validity, structural validity, cross-cultural validity, and different indices of reliability comprising 1) reliability (test-retest, inter-rater, intra-rater), 2) internal consistency, and 3) measurement error.

**Results:**

Of 7570 studies, 170 were eligible, revealing 44 measures of dissociation (86% trait dissociation, 14% state dissociation) and their 14 adapted versions. None of the measures met all COSMIN criteria for good psychometric properties and high methodological quality. Overall, methodological quality was rated as follows: ‘doubtful’ for content validity, ‘adequate’ for measurement error and cross-cultural validity, and ‘very good’ for structural validity and internal consistency. Most included studies did not assess the reliability of investigated measures.

**Conclusion:**

The Dissociative Experiences Scale (DES), adolescent DES, Peritraumatic Dissociative Experiences Questionnaire, Somatoform Dissociation Questionnaire-20, and Cambridge Depersonalisation Scale demonstrated strong evidence for measuring general, child/adolescent, trauma-related (state) or somatoform dissociation, and depersonalisation, respectively. Future research should refine or develop dissociation measures following COSMIN guidelines to ensure robust methodology and psychometric properties.

## Highlights

• We reviewed the properties and quality of existing dissociation measures.
• 44 measures and 14 adapted versions were found, most assessing trait dissociation.
• Only one measure for adolescent dissociation (A-DES) met all COSMIN psychometric and quality criteria.
• The Dissociative Experiences Scale is the strongest measure of trait dissociation.
• Further validation and development of dissociation measures is warranted.

## 1 Introduction

Dissociation is broadly defined as a state of disruption or discontinuity in the integration of consciousness, memory, identity, emotion, perception, body representation, motor control and behaviour (American Psychiatric Association, 2022). This can refer to ‘state dissociation’, as in transient experiences of detachment, identity disturbance or amnesia, but can also encompass the tendency to experience, or vulnerability to develop, such dissociative states, referred to as ‘trait dissociation’ (Giesbrecht et al., 2008). A core feature of dissociative psychopathology is elevated trait dissociation, which is found in dissociative identity disorder, depersonalisation-derealisation disorder, and dissociative amnesia, for example (American Psychiatric Association, 2022). A range of measures have been developed to assess one's degree of, or tendency to experience, dissociation, irrespective of its specific definition (Butler et al., 2019).

Dissociation, particularly dissociative disorders characterised by the experience of pronounced dissociative symptomatology, imposes a significant financial burden on society and the diagnosis and specialised treatment of such conditions can result in reduced service utilisation and associated cost over time (Langeland et al., 2020). The prevalence of dissociative disorders has varied widely, ranging from less than 1%–11.4%, depending on the population, assessment methods, and specific dissociative disorder diagnoses (Kate et al., 2020; Yang et al., 2023). Dissociation is linked to various psychiatric conditions, especially those related to trauma, which is its significant risk factor. Additionally, dissociation can also serve as a coping mechanism against such unbearable and overwhelming experiences (Krause-Utz, 2022). Furthermore, the presence or heightened severity of dissociation can serve as a prognostic indicator in specific psychiatric disorders including borderline personality disorder, post-traumatic stress disorder (PTSD) and functional neurological disorder (Beutler et al., 2022; Campbell et al., 2022; Krause-Utz et al., 2021). In addition, dissociation is increasingly recognised as an important feature of a range of psychiatric symptoms, such as hallucinations and psychosis (Bloomfield et al., 2021; Longden et al., 2020; Pilton et al., 2015). Therefore, appropriate measures for assessing dissociation are beneficial for clinicians and researchers in terms of evaluating dissociation and following treatment outcomes.

Despite the significance of, and clinical need for, robust dissociation measurement, a comprehensive and systematic review of existing measures has not been conducted recently (Cardeña, 2008). Neither the methodological quality, nor the psychometric properties, of such measures has been thoroughly examined. This leaves clinicians and researchers at a standstill regarding the value and rigour of different measures, and which are most well-suited to specific populations and/or dissociative symptoms. Thus, our review aims to address this gap by evaluating the methodological quality and psychometric properties, along with the relative strengths and weaknesses, of existing measures designed to assess state and trait dissociation.

## 2 Method

### 2.1 Search strategy

This review was registered with PROSPERO (CRD42023423485). Preferred Reporting Items for Systematic Reviews and Meta-Analyses (PRISMA) guidelines were followed (Moher et al., 2009), and methodological quality and psychometric properties were assessed using the COnsensus-based Standards for the selection of health Measurement INstruments (COSMIN) criteria (Mokkink et al., 2018; Prinsen et al., 2018; Terwee et al., 2018). A narrative synthesis was conducted to summarise the findings.

In May 2023, three databases (MEDLINE, Embase, and PsycINFO) were systematically searched using comprehensive search terms for dissociation, measurement and psychometric properties (Supplementary Table 1). The search terms were combined using the “AND” operator across all three domains. The searches were performed in the abstract, title, and keywords fields. References and relevant reviews were also searched manually to identify additional sources.

### 2.2 Eligibility

Studies evaluating the psychometric properties of measures specifically designed to assess dissociation, published in peer-reviewed journals or books, were included. We included only measures that quantify the severity of either state or trait dissociation and can be used regardless of any experimental manipulation. There were no restrictions on publication date. Exclusion criteria were: 1) clinical interviews, 2) not published in English, 3) insufficient data or unpublished sources (e.g., doctoral theses, poster presentations), and 4) reviews and meta-analyses.

### 2.3 Study selection and data extraction

SW conducted the deduplication of search results. Two reviewers (SW and XH) independently screened titles and abstracts. Full-text assessment and data extraction (e.g., number of items, response method, response option, scoring range, and representative items) were carried out by SW. Any disagreements during the screening or uncertainties during data extraction were resolved through consultation with senior authors (LSMM or SP).

### 2.4 Quality appraisal and the COSMIN checklist

Two independent raters (SW and XH) completed the methodological quality assessment and psychometric properties evaluation. Cohen's kappa was 0.9, indicating a strong inter-rater reliability. The COSMIN Risk of Bias checklist was used to assess methodological quality and risk of bias of the included studies (Table 1). Each domain was rated as ‘very good’, ‘adequate’, ‘doubtful’, or ‘inadequate’ for all items within that domain. The overall rating for the methodological quality of each domain was determined based on the lowest rating among its items (Mokkink et al., 2018; Terwee et al., 2018). Content validity was rated once as its result is applicable with other studies using identical constructs. Meanwhile, other domains (e.g., structural validity, internal consistency, cross-cultural validity, reliability, and measurement error) were assessed for methodological quality in all included studies.

**Table 1:** COSMIN definitions of relevant measurement properties.

Properties	Explanation
Content validity	Does the measure include items relevant to the construct of interest and covering the full scope of the outcome? Evidence should be presented of an assessment concerning item relevance and scope during the development of each measure.
Structural validity	The items within a scale should be related to each other and can be tested by either exploratory factor analysis (EFA; explain at least 50% of all variances) or confirmatory factor analysis (CFA; factors matched the defined criteria), and Item Response Theory (IRT) with Rasch analysis.
Internal consistency	The items within a scale should tap into the same basic underlying construct and correlate with the overall score in case of the exclusion of that specific item. It can be tested using the value of Kuder-Richardson Formula 20 (KR-20) or Cronbach's alpha ≥0.70, for each subscale.
Reliability	The results remain the same or similar across the appropriate interval (test-retest) or in the rating completed by two equal groups (split-half), measured by the Intraclass correlation coefficient (ICC) ≥ 0.70. It also refers to the stability of participants during their measure rating.

The reported psychometric properties were evaluated and scored as sufficient (+), insufficient (−) or indeterminate (?) based on the criteria for good measurement properties (Prinsen et al., 2018). In addition to evaluating structural validity, internal consistency, and reliability, the assessment also encompassed cross-cultural validity in relevant studies. Specific validity types such as divergent, convergent, and concurrent validity were not included in the COSMIN framework. It is noteworthy that the COSMIN defines ‘reliability’ with a broader concept, comprised of 1) reliability (test-retest, inter-rater or intra-rater), 2) internal consistency, and 3) measurement error (Prinsen et al., 2018). As a result, ‘internal consistency’ was rated separately from reliability.

### 2.5 Levels of evidence

All measures were graded for their level of evidence, considering the consistency of scores for both psychometric properties and methodological quality ratings (Table 2). Methodological quality ratings were coded with ‘+’ or ‘-‘ signs, denoting good or poor properties, respectively. Levels of evidence were categorised as ‘strong’ (+++ or ---), ‘moderate’ (++ or --), ‘limited’ (+ or -), ‘conflicting’ (±), or ‘unknown’ (?) (Terwee et al., 2018).

**Table 2:** Levels of evidence.

Level of evidence	Definition
Strong (+++ or ---)	There are several studies of good methodological quality assessing the measure. The symbol ‘+++’ indicates sufficient evidence of good measurement properties, while ‘---’ denotes insufficient evidence.
Moderate (++ or --)	There are several studies of fair methodological quality or in one study of good methodological quality. The symbol ‘++’ indicates sufficient evidence of good measurement properties, while ‘--’ denotes insufficient evidence.
Limited (+ or -)	There is only one study of fair methodological quality with sufficient evidence of good measurement properties (+) or insufficient (−).
Conflicting (±)	The findings are conflicting.
Unknown (?)	There are only studies of poor methodological quality.

## 3 Results

**Fig. 1:**
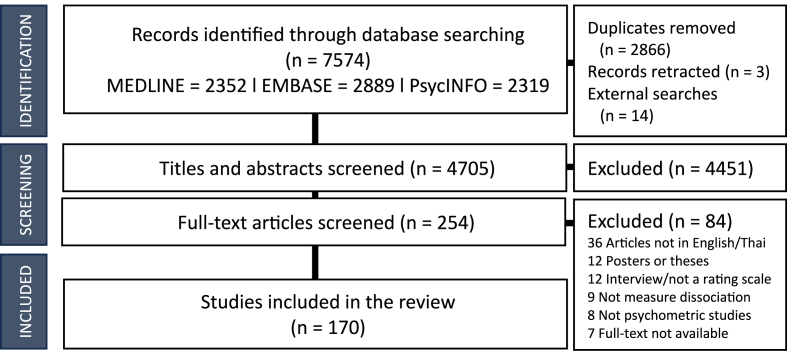
PRISMA flow diagram.

One hundred and seventy studies were eligible for inclusion (Fig. 1). In total, 44 measures of dissociation were identified, and all original articles were included except the *Fewtrell Depersonalisation Scale* (FWS; Fewtrell, 2000) because of its unavailability. The *Tellegen Absorption Scale* (TAS; Tellegen and Atkinson, 1974) was also excluded because it was not conceptualised originally as a measure of dissociation (Terhune and Jamieson, 2021). Although ‘absorption’ can be a marker of dissociative pathology, its definition in the case of the TAS is different to measures of dissociation (i.e., the DES), with the two scales examining different constructs (Simeon et al., 2009; Soffer-Dudek et al., 2015). The *Dissociative Experiences Scale* (DES) had the highest number of translated and validated studies (*n* = 52). All measures used self-rated methods except for five measures that were observer-rated or caregiver-rated: the original *Peritraumatic Experiences Questionnaire* (Marmar et al., 1994), the *Depersonalisation Severity Scale* (DSS; Simeon et al., 2001), the *Child Dissociation Checklist* (CDC; Putnam et al., 1993), the *Child/Adolescent Dissociation Checklist* (CADC; Reagor et al., 1992), and the *Clinician-Administered Dissociative States Scale* (CADSS; Bremner et al., 1998), with the last two also capable of being utilised as self-rated measures. Most measures assessed trait dissociation (38; 86%) whereas only six measures (14%) were designed to assess state dissociation. An overview of included measures can be found in Supplementary Table 2.

The majority of studies were conducted in the United States (40.0%) and were available in English. In addition to studies that examined general dissociation (*n* = 102; 60.0%), we identified studies evaluating measures of child and adolescent dissociation (*n* = 25; 14.7%), depersonalisation (*n* = 15; 8.8%), somatoform dissociation (*n* = 12; 7.1%), and dissociation in PTSD (*n* = 11; 6.5%). Five studies were classified as miscellaneous (2.9%), either focusing on other specific aspects of dissociation (*n* = 4) or incorporating dissociation assessment within non-dissociative questionnaires (*n* = 1). Supplementary Table 3 shows the study characteristics and demographic profiles of participants in all included studies.

### 3.1 Methodological quality and psychometric properties

The methodological quality of all included studies was assessed using the COSMIN checklist. Content validity was rated for 46 articles. Most studies were rated as having doubtful quality (n = 34; 73.9%) and one, eight, and three measures met criteria for inadequate, adequate, and very good methodological quality, respectively.

Structural validity, internal consistency, reliability, and measurement error were assessed in all studies. Approximately half of the studies (*n* = 76; 44.7%) showed very good structural validity. Internal consistency was frequently reported (*n* = 133; 78.2%), and the methodological assessment yielded very good results in this domain for most studies (*n* = 121; 71.2%). In contrast, many studies were indetermined for evidence of good measurement (*n* = 69; 40.6%). Further, more than half of the studies did not assess reliability (*n* = 105; 61.8%), resulting in a high proportion of inadequate methodological quality ratings in this domain (*n* = 98; 57.6%). Adequate grading was most prevalent for the measurement error domain (*n* = 124; 72.9%).

Cross-cultural validity was assessed in studies with different contexts such as translated versions or countries of study locations (*n* = 70). Among the assessed studies, methodological quality of cross-cultural validation was rated as adequate (*n* = 39; 55.7%), very good (*n* = 19; 27.1%), or doubtful (*n* = 12; 17.1%). All assessment results are described in Supplementary Table 4.

### 3.2 Levels of evidence

**Fig. 2:**
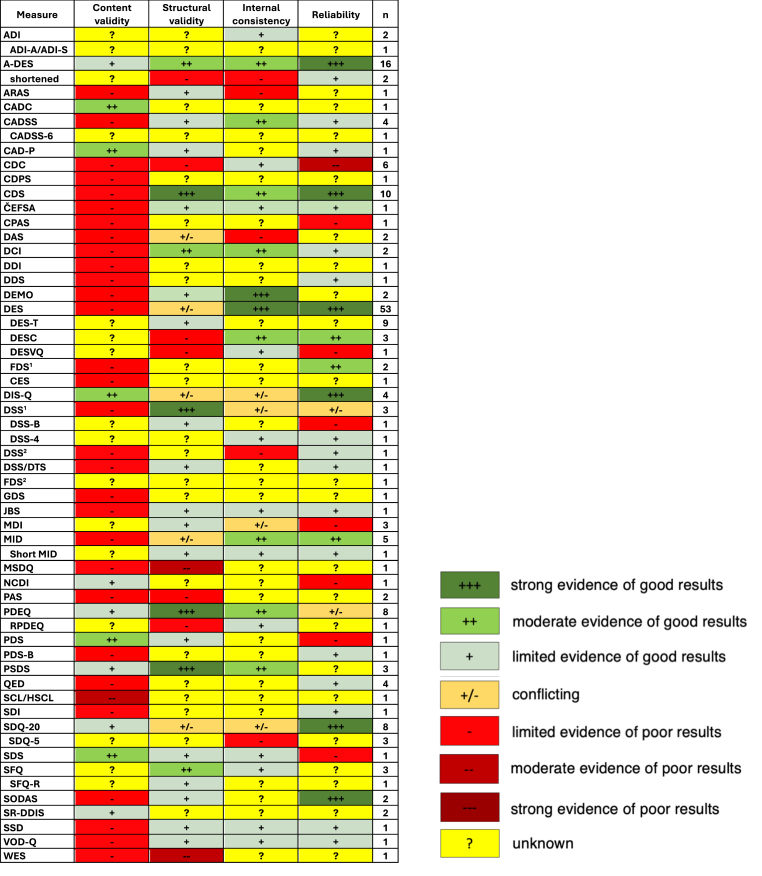
Level of evidence of included measures of dissociation (*k* = 30) ADI – Acute Dissociation Inventory; A-DES – Adolescent Dissociative Experiences Scale; ARAS – Attentional Resource Allocation Scale; CADC – Child/Adolescent Dissociation Checklist; CADSS – Clinician-administered Dissociative States Scale; CAD-P – Cognitive Appraisal of Dissociation in Psychosis; CDC – Child Dissociative Checklist; CDPS – Childhood Dissociative Predictor Scale; CDS – Cambridge Depersonalisation Scale; CEFSA – *Černis* Felt Sense of Anomaly; CPAS – Children's Perceptual Alteration Scale; DAS – Dissociative Ability Scale; DCI – Detachment and Compartmentalisation Inventory; DDI – Depersonalisation-Derealisation Inventory; DDS – Dixon's Depersonalisation Scale; DEMO – Dissociative Experiences Measure, Oxford; DES – Dissociative Experiences Scale; DES-T – Dissociative Experiences Scale-Taxon; DESC – Dissociative Experiences Scale-Comparisons; DESVQ – Dissociative Experiences Scale-Verbal Quantifier; FDS^1^ – Fragebogen zu Dissoziativen Symptomen; CES – Curious Experiences Survey; DIS-Q – Dissociative Questionnaire; DSS^1^ – Dissociative Symptoms Scale; DSS-B – Brief Dissociative Symptoms Scale; DSS^2^ – Depersonalisation Severity Scale; DSS/DTS – Dissoziations-Spannungs-Skala/Dissociative Tension Scale; FDS^2^ – Fewtrell Depersonalisation Scale; GDS – General Dissociation Scale; JBS – Jacobs and Bovasso's Depersonalisation Scale; MDI – Multiscale Dissociation Inventory; MID – Multidimensional Inventory of Dissociation; MSDQ – Medical Somatic Dissociation Questionnaire; NCDI – North Carolina Dissociation Index; PAS – Perceptual Alteration Scale; PDEQ – Peritraumatic Dissociative Experiences Questionnaire; RPDEQ – RAND Peritraumatic Dissociative Experiences Questionnaire; PDS – Phillips Dissociation Scale; PDS-B – Brief Pathological Dissociation Scale; PSDS – Dissociative Subtype of Posttraumatic Stress Disorder Scale; QED – Questionnaire of Experiences of Dissociation; SCL/HSCL – Symptom Checklist/Hopkins Symptom Checklist augmented with dissociation questionnaires; SDI – Somatoform Dissociation Index; SDQ – Somatoform Dissociation Questionnaire; SDS – Subclinical Dissociation Scale; SFQ – Strange-Face Questionnaire; SFQ-R – Strange-Face Questionnaire-revise; SODAS – Scale of Dissociative Activities; SR-DDIS – Self-report Dissociative Disorders Interview Schedule; SSD – State Scale of Dissociation; VOD-Q – Van Obsessional Dissociation Questionnaire; WES – Wessex Dissociation Scale.

Levels of evidence for all measures are shown in Fig. 2. There were 30 measures, and their adaptations, that were assessed by one study for each. Positive results were more common among internal consistency and reliability domains, compared to content validity and structural validity.

The DES was the most evaluated measure and received very strong positive ratings for its internal consistency and reliability. The DES was initially developed as a 28-item self-rated questionnaire with three subscales: 1) amnestic fragmentation of identity, 2) absorption and imaginative involvement, and 3) depersonalisation and derealisation, established through factor analyses (Bernstein and Putnam, 1986; Darves-Bornoz et al., 1999). Certain studies categorised this measure into four factors, adding an additional ‘miscellaneous’ subscale (Tzikos et al., 2021). Conversely, another subscale, the DES-Taxon (DES-T), was derived from the DES by selecting eight items signifying severe dissociative psychopathology, enabling the identification of pathological dissociation, specifically dissociative identity disorder (Spitzer et al., 2015; Waller et al., 1996). Validation studies of the DES encompassed a diverse range of populations, including rape victims (Darves-Bornoz et al., 1999), offenders (Ruiz et al., 2008), non-clinical populations (Boysan, 2014; Chan et al., 2017; Laroi et al., 2013), as well as individuals with psychiatric disorders such as eating disorders (Gleaves and Eberenz, 1995), schizophrenia (Jeong et al., 2021; Oh et al., 2015), and substance use disorder (Gidzgier et al., 2022). The DES has also served as a model for dissociation measurement in other populations, including children and adolescents (Armstrong et al., 1997; Putnam et al., 1993).

Alongside general dissociation measures, the adolescent DES (A-DES; *n* = 16), *Peritraumatic Dissociative Experiences Questionnaire* (PDEQ; *n* = 8), *Somatoform Dissociation Questionnaire-20* (SDQ-20; *n* = 8), and *Cambridge Depersonalisation Scale* (CDS; *n* = 10) exhibited the most satisfactory levels of evidence for measures of child and adolescent dissociation, trauma-related state dissociation, somatoform dissociation, and depersonalisation, respectively. Several studies examined the psychometric properties of more than one measure, resulting in a cumulative evaluation count exceeding the cumulative number of included studies (164), as indicated in Fig. 2.

## 4 Discussion

### 4.1 Overview of assessment of methodological quality and psychometric properties

The aim of this review was to offer comprehensive insights into the methodological quality and psychometric properties of existing measures of dissociation. In all, 44 original measures and 12 adapted versions were identified. However, none of the included measures exhibited psychometric properties and methodological quality that fully aligned with the COSMIN standards. A significant factor in this determination was content validity, for which the majority of measures received an ‘inadequate’ rating.

To achieve a ‘very good’ rating for content validity, as per COSMIN guidelines, a complex methodological approach is necessitated, involving soliciting feedback from both patients and professionals to assess the measure's relevance and comprehensiveness (Mokkink et al., 2018; Terwee et al., 2018). This omission of stakeholder involvement has been evident in evaluations of various health outcome measures adhering to similar criteria (Hoglund et al., 2023; Pellekooren et al., 2021). It is important that the development of future dissociation measures, and or the refinement of existing measures, prioritises input from both professionals and patients to encompass the most comprehensive and relevant constructs, which can subsequently be incorporated as items within the measure.

According to COSMIN guidelines (Prinsen et al., 2018), structural validity evaluation requires confirmatory or exploratory factor analyses. By contrast, numerous studies employed other forms of validity (i.e., convergent, divergent, and concurrent validity), which were not categorised under structural validity. Consequently, this led to lower methodological quality ratings and hindered the attainment of good standards for measurement properties.

For most measures, evidence for good internal consistency was considered ‘indeterminate’. According to the COSMIN, to receive a favourable rating, Cronbach's alpha coefficients for each subscale must meet the set value of ≥0.70 (Mokkink et al., 2018; Terwee et al., 2018). Accordingly, studies reporting only overall Cronbach's alpha coefficients could not be ranked higher than ‘indeterminate’ unless the measure's unidimensionality was adequately established. Researchers are therefore encouraged to thoroughly examine their analyses and effectively present their findings to attain higher levels of grading for reported psychometric properties.

Among the measures under review, reliability consistently garnered the most favourable assessment. The use of either split-half or test-retest reliability warrants at least adequate methodological quality. In the context of test-retest reliability, suitable intervals were identified in most measures (Marx et al., 2003), with the exception of certain measures on child and adolescent dissociation, where the time span ranged from five months to one year (Correia-Santos et al., 2022; Putnam et al., 1993; Reyes-Perez et al., 2005).

#### 4.1.1 Measurement profiles

The item count within the examined measures varied from four to 218 (Dell, 2006; Stiglmayr et al., 2009). Opting for fewer items may enhance respondent acceptability, but it could potentially constrain the scope of certain property evaluations (Levis et al., 2020). This trend aligns with the levels of evidence outlined in our review. Future high-quality studies on measures of dissociation with low numbers of items are necessary to help draw more definitive conclusions. Five measures included in this review contained fewer than eight items, and none of the seven studies assessing these measures reached a satisfactory level of evidence (Kira and Shuwiekh, 2022; Nijenhuis et al., 1997, 1998; Nilsson et al., 2015; Rodrigues et al., 2021; Simeon et al., 2001; Stiglmayr et al., 2009).

A variety of response options were observed across included studies. The predominant approach was employing a Likert-scale with scores ranging from three to 100. Earlier generations of measures utilised different response formats, such as true or false (Phillips, 1994; Reagor et al., 1992; Riley, 1988), or marking a line from 0% to 100% in the original version of the DES (Bernstein and Putnam, 1986). However, the DES was subsequently modified into a 0–100 rating scale (Ellason et al., 1994), making it suitable for use by both patients as raters and clinicians as interpreters.

Based on our findings, the DES was the most widely translated measure (14 languages) and was validated in diverse global contexts. However, similar to other measures, the DES is susceptible to response and experimenter biases (van IJzendoorn & Schuengel, 1996). Notably, none of the studies included in this review were conducted in African countries, where dissociation might exhibit distinct prevalence and conceptualisation (Douglas, 2009; Mianji and Semnani, 2015). Our eligibility criteria limited inclusion to articles published in English, hence studies involving measures in non-English languages within African countries were excluded, leading to the omission of those measures. For instance, we excluded one study from Tunisia (Moyano et al., 2003) which was published in French, which we acknowledge as a limitation in terms of language inclusivity.

Among the various measures assessing general dissociation, child and adolescent dissociation, trauma-related/state dissociation, somatoform dissociation, and depersonalisation/derealisation, the most robust levels of evidence were found for the DES, A-DES, PDEQ, SDQ-20, and CDS, respectively (Armstrong et al., 1997; Bernstein and Putnam, 1986; Marmar et al., 1994; Nijenhuis et al., 1996; Sierra and Berrios, 2000). Each of these measures received the highest ratings in terms of methodological quality and mostly demonstrated moderate to strong evidence of good results, as described in Fig. 2. However, despite the preferrable psychometric properties of the PDEQ for participants with PTSD, this measure has not yet been validated or utilised in populations with PTSD and disturbances in self-organisation, more precise diagnoses of PTSD, or complex PTSD (Hamer et al., 2024).

Thirty-eight measures (86%) were developed to evaluate trait dissociation using retrospective approaches, either with or without limited timeframes. Six measures (14%) were specifically designed to assess state dissociation; four measures (i.e., Acute Dissociation Inventory; ADI, Clinician-Administered Dissociative States Scale; CADSS, State Scale of Dissociation; SSD, and Strange-Face Questionnaire; SFQ) evaluated the level of state dissociation (Bremner et al., 1998; Caputo, 2015; Kruger and Mace, 2002; Leonard et al., 1999), whereas the other two assessed the severity of particular symptoms during a dissociative state, including ‘Felt Sense of Anomaly’ (ČEFSA; Cernis et al., 2021) and ‘Cognitive Appraisal’ (Cernis et al., 2020). All state measures are self-rated, except for the Clinician-administered Dissociative States Scale, which can be either self-rated and/or observer-rated (Bremner et al., 1998). This measure was adapted into a shorter 6-item version for monitoring dissociative effects during ketamine infusion (Rodrigues et al., 2021).

Interestingly, the SFQ appears particularly unique in its approach to assessing state dissociation during face-to-face interpersonal interactions and face-to-face interactions with oneself during mirror staring (Caputo, 2015). Unlike other measures of dissociation that tend to focus on more generalised dissociative experiences or retrospective accounts of dissociation, the SFQ was designed to specifically target transient dissociative experiences in these direct, personal contexts. In contrast, the ADI was originally developed for laboratory settings (Leonard et al., 1999). Participants underwent three dissociation induction exercises (a dot-staring task, an audio/photic stimulation task, and a stimulus deprivation task using a mask and headset without audio and visual input). The ADI assesses the immediate severity of dissociative symptoms with questions such as “How much of the past 10 min do you feel you can recall?”.

### 4.2 Strengths and limitations

This is the first systematic review to assess the quality of measures of dissociation, yielding a substantial number of included studies and a wide range of measures, providing a comprehensive overview of their psychometric properties. New measurement studies, such as the ČEFSA, are included. Although a strength of this review is the utilisation of the COSMIN checklist, which has been well-developed and widely used, several included studies were conducted prior to the guideline's establishment, leading to potential misalignment in quality appraisal. Additionally, studies receiving negative or unknown ratings may have obtained a better rating if they had reported the requisite values for the COSMIN assessment. Therefore, while COSMIN offers valuable insights, it may not comprehensively reflect actual methodological quality or good standards of measurement properties for all included studies, but rather the reporting approach. Certain psychometric properties, such as criterion validity or structural validity, may be evaluated in only a few studies, leading to a lower level of evidence for these domains despite a high number of validation studies. The application of specific dimensions of the COSMIN criteria were tailored for this review. Additionally, the COSMIN, originally developed for patient-rated measures, might not fully capture the considerations for observer- or caregiver-rated measures and its assessment relies on the judgement of raters. Consequently, caution should be exercised when applying the COSMIN to such measures (Bremner et al., 1998; Putnam et al., 1993; Reagor et al., 1992; Rodrigues et al., 2021; Simeon et al., 2001).

We included only measures specifically designed to assess dissociation. Therefore, measures evaluating psychiatric syndromes encompassing dissociation, such as several borderline personality severity measures (Kleindienst et al., 2020; Pfohl et al., 2009), and the Stanford Acute Stress Disorder Questionnaire (Cardena et al., 2000), were excluded. Despite trauma-related dissociative symptoms being assessed, this measure differs from the PDEQ and other included measures, which are specifically designed to assess peri-traumatic dissociative symptoms.

Additionally, we did not include diagnostic interviews in this review. While such measures can provide severity gradings, the continuous measures derived from these interviews are not as comprehensive in terms of scoring range as those computed with the psychometric measures considered here. Furthermore, these interviews require an experienced rater for proper administration and can be time-consuming, with some taking over 1.5 h to complete. Including them would have also shifted the focus of the review towards diagnostic assessments, which was not our original intention.

This review underscores the ongoing need for continued development and refinement of dissociation measures. Considering that the overall methodological quality did not completely meet the COSMIN standard, particularly content validity, future research on psychometric properties should place a special emphasis on addressing this concern. Attention should also be paid to other properties and methodological considerations, while also ensuring adherence to the guidelines outlined by COSMIN.

## 5 Conclusions

This review included 44 different measures of dissociation across 164 studies evaluating their psychometric properties. The methodological quality assessment based on COSMIN guidelines revealed varying levels across studies: content validity was rated as ‘doubtful’, measurement error and cross-cultural validity were ‘adequate’, structural validity and internal consistency were ‘very good’, and reliability was ‘not assessed’. The DES, A-DES, PDEQ, SDQ-20, and CDS demonstrated the highest levels of evidence for measures of general dissociation, child and adolescent dissociation, trauma-related/state dissociation, somatoform dissociation, and depersonalisation, respectively. However, the availability of measures tailored to specific cultural contexts, such as African countries, was limited. Additionally, COSMIN itself may not be entirely objective and is dependent on the subjective judgement of raters. Future measures of dissociation, designed in accordance with COSMIN guidelines, should be developed with the aim of enhancing methodological quality and psychometric properties.

## CRediT authorship contribution statement

**Sorawit Wainipitapong:** Writing – review & editing, Writing – original draft, Methodology, Investigation, Formal analysis, Data curation. **L.S. Merritt Millman:** Writing – review & editing, Supervision, Methodology. **Xi Huang:** Formal analysis, Data curation. **Lillian Wieder:** Writing – review & editing, Methodology. **Devin B. Terhune:** Writing – review & editing, Methodology. **Susannah Pick:** Writing – review & editing, Validation, Supervision, Project administration, Methodology, Conceptualization.

## Funding details

SP and LSMM have been funded by an 10.13039/501100000265MRC Career Development Award to SP (2021–2026 [MR/V032771/1]). SW has been granted by the Thai Red Cross Society.

## Declaration of competing interest

None.
