# Supplementary material for: Assessing dissociation: A systematic review and evaluation of existing measures
Source: J Psychiatr Res. 2025 Jan;181:91–8. doi: 10.1016/j.jpsychires.2024.11.040 (PMC11904123; doi:10.1016/j.jpsychires.2024.11.040)
Supplement: Multimedia component 1 [file mmc1.docx]

**Supplementary table 1 Combined search terms**

| **Domains** | **Search terms** |
| --- | --- |
| 1. Dissociation | exp dissociation/  (dissociati* or dereali*ation or depersonali*ation or multiple personalit* or compartmentali*ation or detachment or possession* or fugue or trance).tw. OR ((psychogenic or dissoc*) ADJ (amnesi* or memory)).tw. |
| 2. Measurement | (scale* or score* or measure* or tool* or questionnaire* or inventor* or checklist or instrument or assessment).tw. |
| 3. Psychometric   properties | MEDLINE, PsycINFO: exp psychometric/ + Embase: exp psychometry/  (reliability or validity or responsiveness or psychometric or consistency).tw,kf. |

**Supplementary table 2 Overview of dissociation measures included in this review**

| **Measure**  **(years)** | **Items,**  ***n*** | **Response**  **method** | **Response**  **option** | **Range**  **of score** | **Language versions** | **Type of dissociation (state vs trait) measured (when applicable), example items and citation of original article** |
| --- | --- | --- | --- | --- | --- | --- |
| **1. General dissociation** | | | | | | |
| 1.1 ADI   (1999) | 35 | Self-rated | 0=everything to 100=nothing | 0-100 | English | State dissociation: 1. How much of the past 10 min do you feel you can recall?  (Cox et al., 1999) |
| ADI-A/  ADI-S | 6 | Self-rated | 0=everything to 100=nothing | 0-100 | English | State dissociation:  1. What was the maximum amount of fear you experienced  during the last 10 min? 2. Did you experience any ‘palpitation’ or racing of your heart?  (Leonard et al., 2000) |
| 1.2 ARAS  (2010) | 15 | Self-rated | 0=never to 4=always | 0-60 | English | Trait dissociation:  1. When I listen to music, I can get so caught up in it that I   don’t notice anything else.  2. The experience of finding myself in a place and having no   idea how I got here. (Carleton et al., 2010) |
| 1.3 CADSS  (1998) | 27 | Self-rated and Observer-rated | 0=not at all to 4=extremely | 0-108 | English  German  French | State dissociation:  1. Do things seem to be moving in slow motion?  2. Do things happen that you later cannot account for?  (Bremner et al., 1998) |
| CADSS-6  (2021) | 6 | Observer-rated | 0=not at all  to 4=extremely | 0-108 | English | (Rodrigues et al., 2021) |
| 1.4 ČEFSA  (2021) | 35 | Self-rated | 0=never to 4=always | 0-140 | English | State dissociation:  1. I feel like a stranger to myself.  2. I feel detached from my emotions. (Cernis et al., 2021) |
| 1.5 DAS  (2013) | 17 | Self-rated | 1=I am unable to do this to 5=I am very capable of doing this | 17-85 | English  Spanish | Trait dissociation:  1. When I am multitasking, I have the ability to process some things without specifically focusing on them.  (Fisher et al., 2013) |
| 1.6 DEMO  (2008) | 30 | Self-rated | 1=not at all to 5=most of the time | 30-150 | English  Hong Kong  Chinese | Trait dissociation:  1. I have the feeling that other people, other things and the world surrounding me are not real.  2. I hear someone talking when no-one nearby has actually said anything. (Cernis et al., 2018) |
| 1.7 DES  (1986) | 28 | Self-rated | 0-100 scale for each item then mean calculation  Original: marking the line from 0%-100%  DES-II (1994): rating score out of 100 | 0-100 | English  Dutch  Turkish  French  German  Finnish  Swedish  Hebrew  Portuguese  Korean  Persian  Italian  Hong Kong  Chinese  Greek | Trait dissociation:  1. Some people have the experience of driving a car and suddenly realising that they don’t remember what has happened during all or part of the trip.  2. Some people find that sometimes they are listening to someone talk and they suddenly realise that they did not hear a part or all of what was said.  3. Some people have the experience of not being sure whether things that they remember happening really did happen or whether they just dreamed them.  3. Some people sometimes find that they hear voices inside their head that tell them to do things or comment on things they are doing.  4. Some people sometimes feel as if they are looking at the world through a fog so that people and objects appear far away or unclear. (Bernstein & Putnam, 1986) |
| DES-T  (1996) | 8 | Self-rated | 0-100 scale | 0-100 | English  German  Finnish  Hong Kong  Chinese | (Waller et al., 1996) |
| DESC   (1999) | 28 | Self-rated | Comparisons: much less to much more than others (11 boxes) | N/A | English | (Wright & Loftus, 1999) |
| DESVQ  (1999) | 28 | Self-rated | Verbal quantifier: never to always (11 boxes) | N/A | English | (Wright & Loftus, 1999) |
| FDS^1^ (1998) | 44 | Self-rated | 0-100 scale | 0-100 | German | (Freyberger et al., 1998) |
| CES (1999) | 31 | Self-rated | 1=this never happens to me to 5=this is almost always happening to me | 31-155 | English | (Goldberg & Bernstein, 1999) |
| 1.8 DIS-Q  (1993) | 69 | Self-rated | 1=not at all to 5=extremely | 69-345 | Dutch  Swedish  Japanese | Trait dissociation:  1. I have the feeling that my body is not (really) mine.  2. It happens that I catch myself day-dreaming.  (Vanderlinden et al., 1993) |
| 1.9 DSS^1^  (2018) | 20 | Self-rated | 0=not at all to 4=extremely | 0-80 | English  Korean  Italian | Trait dissociation:  1. I felt like I wasn’t myself.  2. I found myself staring into space and thinking of nothing.  (Carlson et al., 2018) |
| DSS-B  (2022) | 8 | Self-rated | 5 boxes (not at all to more than once a day | N/A | English | (Macia et al., 2022) |
| DSS-4  (2009) | 4 | Self-rated | 0=not very present to 9=very strong | 0-36 | German | (Stiglmayr et al., 2009) |
| 1.10 GDS  (2003) | 15 | Self-rated | 1=not at all to 4=very much so | 15-60 | English | Trait dissociation:  1. My detachment could occur even when I am not drinking, taking drugs or taking medication. (Sapp & Hitchcock, 2003) |
| 1.11 MDI  (2002) | 30 | Self-rated | 1=never to 5 =very often | 30-150 | English | Trait dissociation:  1. Not paying attention because you were in your own world.  2. Suddenly things around you not feeling real or familiar.  (Briere, 2002) |
| 1.12 MID  (2006) | 218 | Self-rated | 0-10 Likert scale then mean calculation | 0-10 | English  Hebrew | Trait dissociation:  1. Forgetting where you put something.  2. Noticing the presence of a child inside you. (Dell, 2006) |
| Short MID  (2021) | 60 | Self-rated | 0=never to 10=always | 0-60 | English | (Kate et al., 2021) |
| 1.13 NCDI  (1995) | 16 | Self-rated | N/A | 0-16 | English | Trait dissociation:  1. At times I have fits of laughing and crying and I cannot control. (Mann, 1995) |
| 1.14 PAS  (1986) | 27 | Self-rated | 1=never to 4=almost always | 27-108 | English | Trait dissociation:  1. I find myself doing things without knowing why.  2. I find my mind blank. (Sanders, 1986) |
| 1.15 PDS  (1994) | 20 | Self-rated | True / False | 0-20 | English | Trait dissociation:  1. Someone has been trying to influence my mind. (Phillips, 1994) |
| 1.16 PDS-B  (2022) | 5 | Self-rated | 0=never happens to 4=often, with little respite | 0-20 | Arabic  English | Trait dissociation:  1. Do you occasionally imagine or hear an inner voice that condemn you, insult you, or direct you to punish yourself or want you to die? (Kira & Shuwiekh, 2022) |
| 1.17 QED  (1988) | 26 | Self-rated | True / False | 0-26 | English | Trait dissociation:  1. I often feel that I am removed from my thoughts and actions. (Riley, 1988) |
| 1.18 SODAS  (2003) | 35 | Self-rated | 1=never to 5=very frequently | 35-175 | English  Turkish | Trait dissociation:  1. I have difficulty staying mentally engaged when I participate in routine tasks. (Mayer & Farmer, 2003) |
| 1.19 SR-DDIS  (2016) | N/A | Self-rated | N/A | N/A | English  Hong Kong  Chinese | N/A  (Ross & Browning, 2016) |
| 1.20 SSD  (2002) | 58 | Self-rated | Not at all to very much so (10 boxes) | N/A | English | State dissociation:  1. Right now things around me seem unreal or dreamlike. (Krüger & Mace, 2002) |
| 1.21 SDS  (2020) | 37 | Self-rated | 1=never to 5=almost all the time | -11 to 140 | Japanese | Trait dissociation:  1. I do not hear things that are being said by people right in front of me. (Masuda et al., 2020) |
| 1.22 SFQ  (2015) | 19 | Self-rated | 0=never/not at all to 4=very often/ extremely | 0-72 | English | State dissociation:  1. Did you see that some facial traits were deformed?  2. Did you see a luminous face?  3. Did you see the face of one of your relatives? (Caputo, 2015) |
| SFQ-R  (2023) | 34 | Self-rated | 0=no/never to 4=very often | 0-132 | English | State dissociation:  1. Did you see the face of a non-human being, like some kind of alien? (Caputo, 2023) |
| 1.23 WES  (2004) | 40 | Self-rated | 0=never to 5=all the time | 0-200 | English | Trait dissociation:  1. Unwanted images from my past come into my head.  (Kennedy et al., 2004) |
| **2. Child and adolescent populations** | | | | | | |
| 2.1 A-DES  (1997) | 30 | Self-rated | 0-10 Likert scale | 0-300 | English  Turkish  Swedish  Korean  Japanese  Czech  Italian  Portuguese | Trait dissociation:  1. I get so wrapped up in watching TV, reading, or playing video games that I don’t have any idea what’s going on around me.  2. I find myself someplace and don’t remember how I got here.  3. My body feels as if it doesn’t belong to me.  (Armstrong et al., 1997) |
| shortened  (2004) | 8 | Self-rated | 0-10 Likert scale | 0-80 | Spanish  Finnish | (Martinez-Taboas et al., 2004) |
| 2.2 CADC  (1992) | 17 | Self-rated  or Caregiver-rated | Yes / No or Unsure | N/A | English | Trait dissociation:  1. Family history of multiple personality or other dissociative disorder: may not have been formally diagnosed as such.  2. Sexual abuse: rape, attempted rape, or unwanted sexual touching or fondling. (Reagor et al., 1992) |
| 2.3 CDC  (1993) | 20 | Observer-rated | 0=not true to 2=very true | 0-40 | English  Turkish  Spanish | Trait dissociation:  1. Child does not remember or denies traumatic or painful experiences that are known to have occurred.  2. Child has two or more distinct and separate personalities that take control over the child's behaviour. (Putnam et al., 1993) |
| 2.4 CDPS  (1992) | 14 | Self-rated | 0=never to 4=very often (almost every day) | 0-56 | English | Trait dissociation:  1. When you were a child, how often did your parents let you know they were pleased with you?  (Branscomb & Fagan, 1992) |
| 2.5 CPAS  (1992) | 28 | Self-rated | 1=never happening to 4=happening all the time | 28-112 | English | Trait dissociation:  1. When I’m awake, I feel like I’m dreaming.  2. I feel like I’m somebody else watching me.  (Evers-Szostak & Sanders, 1992) |
| **3. Trauma-related dissociation** | | | | | | |
| 3.1 PDEQ  - Rater  (1994) | 8 | Observer-rated | 0=in adequate information to 3=threshold | 0-24 | English | Trait dissociation:  1. Moment of losing track or blanking out.  2. Found self acting on automatic pilot.  3. Not aware of things that happened. (Marmar et al., 1994) |
| PDEQ  (1997) | 10 | Self-rated | 1=not at all to 5=extremely true | 10-50 | English  French  Portuguese  Persia  Chilean  Spanish | Trait dissociation:  1. I “blanked out” or “spaced out” or in some way felt that I was not part of what was going on.  2. I felt separate or disconnected from my body or like my body was unusually large or small.  3. There were moments when I wasn’t sure about where I was or what time it was. (Marmar et al., 1997) |
| RPDEQ  (2002) | 8 | Self-rated | 1=not at all to 5=extremely true | 8-40 | English | (Marshall et al., 2002) |
| 3.2 PSDS  (2015) | 15 | Self-rated | Frequency past month: 1=once or twice to 4=daily or almost every day  Intensity: 1=not very strong to 5=extremely strong | N/A | English  Turkish | Trait dissociation:  1. Have there ever been times when you felt disconnected from your body, as if your body were not your own?  2. Have there ever been times when the world around you (other people, objects, places) did not seem real?  3. Have you ever thought that you should be able to remember more about __________ (fill in with participant’s “worst” traumatic event) than you do? (Wolf et al., 2017) |
| **4. Somatic dissociation** | | | | | | |
| 4.1 DSS/DTS  (2010) | 21 | Self-rated | 0-100 scale | N/A | Suspected  German | Trait dissociation:  1. I could not feel my body or parts of my body.  2. I perceived my breathing as having changed.  (Stiglmayr et al., 2010) |
| 4.2 MSDQ  (2019) | 30 | Self-rated | 0=nothing to 4=extremely | 0-120 | Hebrew  Arabic | Trait dissociation:  1. You feel you need more attention.  2. Stomach ache. (Daphna-Tekoah et al., 2019) |
| 4.3 SDI  (2001) | 27 | Self-rated | N/A | N/A | English | Trait dissociation:  1. Frequently notice my hand shakes when I try to do something. (Leavitt, 2001) |
| 4.4 SDQ-20  (1996) | 20 | Self-rated | 1=not applicable to 5=highly applicable | 20-100 | Dutch  German  Turkish  French  Portuguese  Swedish  Spanish | Trait dissociation:  1. It is as if my body, or a part of it, has disappeared.  2. I hear sounds from nearby as if they come from far away.  3. My body, or a part of it, feels numb.  (Nijenhuis et al., 1996) |
| SDQ-5  (1997) | 5 | Self-rated | 1=not applicable to 5=highly applicable | 5-25 | Dutch  Swedish | (Nijenhuis et al., 1997) |
| **5. Depersonalisation/derealisation** | | | | | | |
| 5.1 CDS  (2000) | 29 | Self-rated | Frequency: 0=never to 4=all the time  Duration: 1=few seconds to 6=more than a week | 0-290 | English  Japanese  Italian  Spanish  Greek | Trait dissociation:  1. Out of the blue, I feel strange, as if I were not real or as if I were cut off from the world.  2. Previously familiar places look unfamiliar, as if I had never seen them before. (Sierra & Berrios, 2000) |
| 5.2 DDI  (2002) | 28 | Self-rated | 0=does not occur to 4=very severe | 0-112 | English | Trait dissociation:  1. Surroundings seem strange or unreal.  2. Feel “spacy” or “spaced out”. (Cox & Swinson, 2002) |
| 5.3 DDS  (1963) | 43 | Self-rated | Yes or No with rating from 0, 1, 2, 5, 10, 25, 50, 100, and 1000 | N/A | English | Trait dissociation:  1. Other people seemed changed or unfamiliar.  2. Things that I had been used to now began to seem strange. (Dixon, 1963) |
| 5.4 DSS^2^  (2001) | 6 | Observer-rated | 0=none to 3=severe | 0-18 | English | Trait dissociation:  1. Have you felt unreal or like a stranger to yourself? (Simeon et al., 2001) |
| 5.5 FDS^2^  (2000) | 35 | Self-rated | 0-4 Likert scale | 0-140 | English | Trait dissociation:   1. When I talk about myself, I feel as if I am talking about someone else. (Fewtrell, 2000) |
| 5.6 JBS  (1992) | 32 | Self-rated | 0=never to 4=daily, at least once a day | 0-128 | English | Trait dissociation:  1. When I am interacting with a person or doing an activity, I   must tell myself that I am an interacting with the person or   doing in order to experience it. (Jacobs & Bovasso, 1992) |
| **6. Miscellaneous** | | | | | | |
| 6.1 CAD-P  (2020) | 13 | Self-rated | 0=never to 4=always | 0-52 | English | State dissociation:  1. I don’t look right to other people right now.  2. I can’t trust my own mind. (Cernis et al., 2020) |
| 6.2 DCI  (2019) | 22 | Self-rated | 0=never happened to 7=happens daily | 0-154 | English  Spanish | Trait dissociation:  1. When listening to someone talk, I suddenly realise I do not hear part or all of what was said. (Butler et al., 2019) |
| 6.3 SCL/HSCL  (1990) | 14 | Self-rated | 0,1=not at all to 4=extremely (depended on the original tests that SCL implemented) | 0-56 or 14-56 | English | Trait dissociation:  1. Feeling outside of your body.  2. Things feeling unreal.  3. Feeling disconnected from yourself.  4. A feeling of being far away. (Briere & Runtz, 1990) |
| 6.4 VOD-Q  (2018) | 45 | Self-rated | 11-point scales from 0=never to 100=always | N/A | Turkish | N/A (Boysan et al., 2018) |

ADI – Acute Dissociation Inventory; ADI-A – Acute Dissociation Inventory (subjective anxiety); ARAS – Attentional Resource Allocation Scale; CADSS – Clinician-administered Dissociative States Scale; CEFSA – Černis Felt Sense of Anomaly; DAS – Dissociative Ability Scale; DEMO – Dissociative Experiences Measure, Oxford; DES – Dissociative Experiences Scale; DES-T – Dissociative Experiences Scale-Taxon; DESC – Dissociative Experiences Scale-Comparisons; DESVQ – Dissociative Experiences Scale-Verbal Quantifier; FDS^1^ – Fragebogen zu Dissoziativen Symptomen; CES – Curious Experiences Survey; DIS-Q – Dissociative Questionnaire; DSS^1^ – Dissociative Symptoms Scale; DSS-B – Brief Dissociative Symptoms Scale; GDS – General Dissociation Scale; MDI – Multiscale Dissociation Inventory; MID – Multidimensional Inventory of Dissociation; NCDI – North Carolina Dissociation Index; PAS – Perceptual Alteration Scale; PDS – Phillips Dissociation Scale; PDS-B – Brief Pathological Dissociation Scale; QED – Questionnaire of Experiences of Dissociation; SODAS – Scale of Dissociative Activities; SR-DDIS – Self-report Dissociative Disorders Interview Schedule; SSD – State Scale of Dissociation; SDS – Subclinical Dissociation Scale; SFQ – Strange-Face Questionnaire; SFQ-R – Strange-Face Questionnaire-revise; WES – Wessex Dissociation Scale; A-DES – Adolescent Dissociative Experiences Scale; CADC – Child/Adolescent Dissociation Checklist; CDC – Child Dissociative Checklist; CDPS – Childhood Dissociative Predictor Scale; CPAS – Children’s Perceptual Alteration Scale; PDEQ – Peritraumatic Dissociative Experiences Questionnaire; RPDEQ – RAND Peritraumatic Dissociative Experiences Questionnaire; PSDS – Dissociative Subtype of Posttraumatic Stress Disorder Scale; DSS/DTS – Dissoziations-Spannungs-Skala/Dissociative Tension Scale; MSDQ – Medical Somatic Dissociation Questionnaire; SDI – Somatoform Dissociation Index; SDQ – Somatoform Dissociation Questionnaire; CDS – Cambridge Depersonalisation Scale; DDI – Depersonalisation-Derealisation Inventory; DDS – Dixon’s Depersonalisation Scale; DSS^2^ – Depersonalisation Severity Scale; FDS^2^ – Fewtrell Depersonalisation Scale; JBS – Jacobs and Bovasso’s Depersonalisation Scale; CAD-P – Cognitive Appraisal of Dissociation in Psychosis; DCI – Detachment and Compartmentalisation Inventory; SCL/HSCL – Symptom Checklist/Hopkins Symptom Checklist augmented with dissociation questionnaires; VOD-Q – Van Obsessional Dissociation Questionnaire

**Supplementary table 3 - Study characteristics and demographic profiles of participants**

| **Study ID** | **TEST2** | **TEST3** | **Title** | **Authors** | **Publication years** | **Country** | **Language** | **Populations** | **Total sample sizes** | **Total mean age +/- SD** | **Sample sizes and age:** | **Clinical Sample size** | **Clinical Age +/- SD** | **Non-clinical / control Sample size** | **Non-clinical / control Age +/- SD** | **% of female participants** |
| --- | --- | --- | --- | --- | --- | --- | --- | --- | --- | --- | --- | --- | --- | --- | --- | --- |
| A-DES01 |  |  | Development and Validation of a Measure of Adolescent Dissociation: The Adolescent Dissociative Experiences Scale | Armstrong J et al. | 1997 | USA | English | Clinical - dissociative disorders/experiences; Clinical - non-dissociative disorders; Non-clinical / control | 102 | 14.9 |  | 85 | N/A | 17 | N/A | 45.1 |
| A-DES02 |  |  | Reliability and Validity of the Adolescent Dissociative Experiences Scale | Smith S and Carlson E. | 1996 | USA | English | Non-clinical / control | 60 | 14.6 |  |  |  |  |  | N/A |
| A-DES03 |  |  | The Adolescent Dissociative Experiences Scale: Psychometric Properties and Difference in Scores Across Age Groups | Farrington A et al. | 2001 | UK | English | Non-clinical / control | 768 | N/A |  |  |  |  |  | 49.10% |
| A-DES04 |  |  | Reliability and validity of the Turkish version of the adolescent dissociative experiences scale | Zoroglu et al. | 2002 | Turkey | Turkish | Clinical - dissociative disorders/experiences; Clinical - non-dissociative disorders; Non-clinical / control | 331 | 16.0 +/- 1.32 |  | 130 | 15.9 | 201 | 16.1 +/- 1.2 | 58.60% |
| A-DES05 | CDC2001 |  | Multimodal Assessment of Dissociation in Adolescents: Inpatients and Juvenile Sex Offenders | Friedrich W et al. | 2001 | USA | English | Clinical - dissociative disorders/experiences; Clinical - non-dissociative disorders; Clinical - other disorders / higher vulnerability | Study 1: 70 (adolescent sex offenses)  Study 2: 47 (short-term acute IPD patients) | Study 1: 15.9 +/- 1.5    Study 2: 15.1 +/- 1.6 |  |  |  |  |  | Study 1: 0%    Study 2: 53.2% |
| A-DES06 |  |  | A validation study of the Adolescent Dissociative Experiences Scale | Seeley S et al. | 2004 | USA | English | Clinical - non-dissociative disorders; Non-clinical / control | 65 (normal female and Clinical/sexually abused) |  |  | 31 | 13.6 +/- 1.8 | 34 | 14 +/- 2.2. | 100% |
| A-DES07 |  |  | Dissociation Among Swedish Adolescents and the Connection to Trauma An Evaluation of the Swedish Version of Adolescent Dissociative Experience Scale | Nilsson D and Svedin C | 2006 | Sweden | Swedish | Clinical - other disorders / higher vulnerability; Non-clinical / control | 141 | 15.4 |  | 20 (sexually and physically abused) | 17 | 121 | 15.1 +/- 1.9 | 53.2% (Clinical 90% Control 47.1%) |
| A-DES08 |  |  | The Korean Version of the Adolescent Dissociative Experience Scale: Psychometric Properties and the Connection to Trauma among Korean Adolescents | Shin J et al. | 2009 | Korea | Korean | Clinical - other disorders / higher vulnerability; Non-clinical / control | 404 | 14.9 |  | 33 | 14.6 +/- 1.9 | 371 | 14.9 +/- 1.8 | 49.50% |
| A-DES09 |  |  | Psychometric Properties of the Adolescent Dissociative Experiences Scale (A-DES) in Japanese Adolescents from a Community Sample | Yoshizumi T et al. | 2010 | Japan | Japanese | Non-clinical / control | 2272 | 14.2 +/- 2.3 |  |  |  |  |  | 53.20% |
| A-DES10 |  |  | Dissociation in non-clinical and clinical sample of Czech adolescents. Reliability and validity of the Czech version of the Adolescent Dissociative Experiences Scale | Soukup J et al. | 2010 | Czech | Czech | Clinical Non-clinical / control | 815 | 15.9 |  | 162 | 15.2 +/- 2.3 | 653 | 16.1 +/- 1.9 | 53.3%  (Clinical 73.5% Non clin 48.2%) |
| A-DES11 |  |  | Psychometric properties of the Adolescent Dissociative Experiences Scale in a sample of Italian adolescents | Schimmenti A | 2015 | Italy | Italian | Non-clinical / control | 1806 (high school students) | 16.2 +/- 1.6 |  |  |  |  |  | 58.40% |
| A-DES12 |  |  | Validation of the Italian version of the dissociative experience scale for adolescents and young adults | Da Pasquale et al. | 2016 | Italy | Italian | Non-clinical / control | 633 | 18.2 +/- 3.1 |  |  |  |  |  | 55.50% |
| A-DES13 |  |  | Validation of the Factor Structure of the Adolescent Dissociative Experiences Scale in a Sample of Trauma-Exposed Detained Youth | Kerig et al. | 2016 | USA | English | Clinical - other disorders / higher vulnerability | 784 | 16.1 +/- 1.3 |  |  |  |  |  | 26.30% |
| A-DES14 |  |  | The psychometric properties of the adolescent  dissociative experiences scale (A-DES) in a sample  of Portuguese at-risk adolescents | Correia-Santos P et al. | 2022 | Portugal | Portuguese | Clinical - other disorders / higher vulnerability | 402 | 15.7 +/- 1.2 |  |  |  |  |  | 60.20% |
| A-DES15 |  |  | Dissociation: Factor Structure and the Role of Trauma Among Treatment-Seeking Adolescents | Kyte D et al. | 2023 | USA | English | Clinical - non-dissociative disorders | 1157 | 15.7 +/- 1.5 |  |  |  |  |  | 55.50% |
| A-DES16  (shortended  A-DES) |  |  | The Psychometric Properties of a Shortened Version of the Spanish Adolescent Dissociative Experiences Scale | Martínez-Taboas A eet al. | 2004 | Peurto Rico | Spanish | Non-clinical / control | 459 | N/A |  |  |  |  |  | 29.40% |
| A-DES17  (abbreviated  A-DES) |  |  | Factor Structure, Measurement Invariance, and Abbreviated Versions of The Adolescent Dissociative Experiences Scale (A-DES) | Lindfors K et al. | 2022 | Finland | Finnish | Non-clinical / control | 4072 | 16.1 +/- 1.5 |  |  |  |  |  | 53.90% |
| ARAS01 |  |  | The Attentional Resource Allocation Scale (ARAS): Psychometric Properties of a Composite Measure for Dissociation and Absorption | Carleton R et al. | 2010 | Canada | English | Non-clinical / control | 1074 (Sample 1 = 841; Sample 2 = 233) | 23.2 (Sample 1 = 21.2 +/- 4.1; Sample 2 = 30.3 +/- 11.1) |  |  |  |  |  | 77.7%  (S 1 = 75.5%;  S 2 = 86%) |
| CADC01 |  |  | A Checklist for Screening Dissociative Disorders in Children and Adolescents | Reagor P et al. | 1992 | USA | English | Clinical - dissociative disorders/experiences; Clinical - non-dissociative disorders; Non-clinical / control | Study 1: 115 Children  Study 2: 115 Children and 54 therapists | Study 1: Median 12.0 and Study 2: N/A |  |  |  |  |  | 58.30% |
| CAD-P01 |  |  | Cognitive appraisals of dissociation in psychosis: a new brief measure | Černis E et al. | 2021 | UK | English | Clinical - non-dissociative disorders; Non-clinical / control | Phase 1: 1615  Phase 2: 8287  Clinical: 1026 (Schizophrenia spectrum disorders) | Phase 1: 49.9 +/- 14.4  Phase 2: 45.8 +/- 14.9  Clinical: 41.5 +/- 12.3 |  |  |  |  |  | Phase 1:  88.7%  Phase 2:  85.7%  Clinical: 29.2% |
| CADSS01 |  |  | Measurement of Dissociative States with the Clinician-Administered Dissociative States Scale (CADSS) | Bremner J et al. | 1998 | USA | English | Clinical - dissociative disorders/experiences; Clinical - non-dissociative disorders; Non-clinical / control | 124 | N/A |  |  |  |  |  | 4.00% |
| CADSS02 |  |  | State and Trait Dissociation: Evaluating Convergent and Discriminant Validity | Condon L and Lynn S | 2014 | USA | English | Non-clinical / control | 214 | 19.7 +/- 1.6 |  |  |  |  |  | 53.30% |
| CADSS03 |  |  | A simplified 6-Item clinician administered dissociative symptom scale (CADSS-6) for monitoring dissociative effects of sub-anesthetic ketamine infusions | Rodrigues N et al. | 2021 | N/A | English | Clinical - non-dissociative disorders | 260 | 45.7 |  |  |  |  |  | 55.80% |
| CADSS04 |  |  | The Clinician-Administered Dissociative States Scale (CADSS): Validation of the German Version | Mertens Y and Daniels J. | 2021 | The Netherlands | German | Clinical - dissociative disorders/experiences; Clinical - non-dissociative disorders | 105 | 43.6 +/- 11.5 |  |  |  |  |  | 81.90% |
| CADSS05 |  |  | Inhibition, Attentional Control and Binding Abilities in Relation to Dissociative Symptoms Among PTSD Patients | Vancappel A et al. | 2023 | France | French | Clinical - dissociative disorders/experiences; Clinical - non-dissociative disorders | 83 | 36.2 +/- 13.6 |  |  |  |  |  | 83.10% |
| CDC01 |  |  | Development, reliability, and validity of a child dissociation scale | Putnam et al. | 1993 | USA | English | Clinical - dissociative disorders/experiences; Clinical - other disorders / higher vulnerability; Non-clinical / control | 181 | 10.5 |  | 114 | 10.4 | 67 | 10.7 +/- 3.0 | 86.2% (Control 100% Clinical 78.1%) |
| CDC02 |  |  | The Child Dissociative Checklist | Wherry J et al. | 1994 | USA | English | Non-clinical / control | Study 1 = 73 (OPD) and Study 2 = 26 (IPD) | Study 1 = N/A and Study 2 = N/A |  |  |  |  |  | Study 1 = 29% and Study 2 = 4% |
| CDC03 |  |  | Reliability and Validity of the Turkish Version of the Child Dissociative Checklist | Zoroglu S et al. | 2002 | Turkey | Turkish | Clinical - dissociative disorders/experiences; Clinical - non-dissociative disorders; Non-clinical / control | 204 | 9.5 |  | 116 | 9.8 | 88 | 9.1 +/- 0.9 | 45.1%  (Clinical 39.7% Non clin 52.3%) |
| CDC04 |  |  | Dissociative Experiences in Children with Abuse Histories: A Replication in Puerto Rico | Reyes-Pérez C et al. | 2005 | Puerto Rico | Spanish | Clinical - other disorders / higher vulnerability | 31 | 8.5 +/- 1.8 |  |  |  |  |  | 51.60% |
| CDC05 |  |  | Pathological Dissociation as Measured by the Child Dissociative Checklist | Wherry J et al. | 2009 | USA | English | Clinical - other disorders / higher vulnerability | 232 | 10.0 +/- 1.7 |  |  |  |  |  | 61% |
| CDPS01 |  |  | Development and Validation of a Scale Measuring Childhood Dissociation in Adults: the Childhood Dissociative Predictor Scale | Branscomb L and Fagan J. | 1992 | USA | English | Clinical - non-dissociative disorders; Non-clinical / control | 161 | 41.1 +/- 10 |  | 95 |  | 66 |  | 13.7%  33% for Normal 0% for Patient |
| CDS01 |  |  | The Cambridge Depersonalisation Scale: a new instrument for the measurement of depersonalisation | Sierra M and Berrios G | 2000 | UK | English | Clinical - dissociative disorders/experiences; Clinical - other disorders / higher vulnerability | 77 | 34 +/- 10.2 |  |  |  |  |  | 50% |
| CDS02 |  |  | Unpacking the depersonalization syndrome: an exploratory factor analysis on the Cambridge Depersonalization Scale | Sierra M et al. | 2005 | UK | English | Clinical - dissociative disorders/experiences | 138 | 35.5 +/- 11.9 |  |  |  |  |  | 43.5 |
| CDS03 |  |  | Reliability and validity of a Japanese version of the Cambridge depersonalization scale as a screening instrument for depersonalization disorder | Sugiura M et al. | 2009 | Japan | Japanese | Clinical - dissociative disorders/experiences;  Healthy control | 59 | 25.9 +/- 7.5 |  |  |  |  |  | 66.10% |
| CDS04 |  |  | Italian (cross cultural) adaptation and validation of the Cambridge Depersonalization Scale (CDS) | Migliorini V et al. | 2011 | Italy | Italian | Clinical - non-dissociative disorders | 92 | 36.5 +/- 14.6 |  |  |  |  |  | 60.90% |
| CDS05 | MDI2012 | DES2012 | Construct Validity of Three Depersonalization Measures in Trauma-Exposed College Students | Blevins C et al. | 2012 | USA | English | Clinical - other disorders / higher vulnerability | 209 | 19.9 +/- 2.0 |  |  |  |  |  | 60.30% |
| CDS06 |  |  | Factor Structure of the Cambridge Depersonalization Scale in Trauma-Exposed College Students | Blevins C et al. | 2013 | USA | English | Clinical - other disorders / higher vulnerability | Sample1 = 260; Sample2 = 274 | Sample 1 = 20.2 +/- 2.5; Sample 2 = 20.0 +/- 1.9 |  |  |  |  |  | 66.70% |
| CDS07 |  |  | Psychometric Properties of the Cambridge Depersonalization Scale in Puerto Rico | Aponte-Soto M et al. | 2014 | Puerto Rico | Spanish | Non-clinical / control | 300 | 40 +/- 14.3 |  |  |  |  |  | 50% |
| CDS08 |  |  | Depersonalization: An exploratory factor analysis of the Italian version of  the Cambridge Depersonalization Scale | Fagioli F et al. | 2015 | Italy | Italian | Clinical - non-dissociative disorders | 149 | 32.4 +/- 14.6 |  |  |  |  |  | 58.40% |
| CDS09 |  |  | Reliability, validity, and psychometric properties of the Greek translation of the Cambridge Depersonalization Scale (CDS) | Kontoangelos K et al. | 2016 | Greece | Greek | Clinical - other disorders / higher vulnerability; Non-clinical / control | 294 (128 HIV OPD patients and 166 controls) |  |  | 128 | 37.1 +/- 9.1 | 166 | 32.4 +/- 13.4 | N/A |
| CDS10 |  |  | Dissociative symptoms as measured by the Cambridge Depersonalization Scale in patients with a bipolar disorder | Tuineag M et al. | 2020 | Canada | English | Clinical - non-dissociative disorders | 73 | 46.3 +/- 11 |  |  |  |  |  | 46.60% |
| CEFSA01 |  |  | A new perspective and assessment measure for common dissociative experiences: ‘Felt Sense of Anomaly’ | Černis E et al. | 2021 | UK | English | Clinical - non-dissociative disorders; Non-clinical / control | Part One - 8861 via Online / Part Two - 1038 Psychosis | Part One - Normal = 40.1 / |  | 1031 | 41.54 +/- 12.3 | 8861 | 40.1 +/- 15.8 | Part One - 86.5% / Part Two - 29.4% |
| CPAS01 |  |  | The Children's Perceptual Alteration Scale (CPAS): A Measure of Children's Dissociation | Evers-Szostak M and Sanders S. | 1992 | USA | English | Clinical - non-dissociative disorders; Non-clinical / control | 53 | Ages of 8-12 years |  | 21 |  | 32 |  | 45.3 |
| DAS01 |  |  | Principal component analysis of a measure of non-pathological dissociation: the dissociative ability scale | Fisher W et al. | 2013 | USA | English | Non-clinical / control | 200 | N/A |  |  |  |  |  | 63% |
| DAS02 |  |  | Factor structure and reliability of the Spanish  version of the Dissociative Ability Scale | Pérez-Fabello M and Campos A. | 2017 | Spain | Spanish | Non-clinical / control | 204 | 22.7 +/- 2.4 |  |  |  |  |  | 53.40% |
| DCI01 |  |  | The Detachment and Compartmentalization Inventory (DCI): An assessment tool for two potentially distinct forms of dissociation | Butler C et al. | 2019 | New Zealand, Australia, South Africa, USA, UK | English | Clinical - dissociative disorders/experiences; Clinical - non-dissociative disorders; Non-clinical / control | 194 | 32.3 |  | 105 | 40.9 +/- 13.4 | 89 | 22.2 +/- 3.8 | 80.90% |
| DCI02 |  |  | Spanish validation of the Detachment and  Compartmentalization Inventory (DCI) in a community and clinical sample. A new instrument for measuring dissociation | Perona-Garcelán et al. | 2020 | Spain | Spanish | Clinical - dissociative disorders/experiences; Clinical - non-dissociative disorders; Non-clinical / control | 394 | 31.6 |  | 40 | 32.6 +/- 12.8 | 268 | 31.5 +/- 13.4 | 57.8% (Clinical 67.5% Control 56.3%) |
| DDI01 |  |  | Instrument to assess Depersonalisation-derealisation in Panic Disorder | Cox B and Swinson R. | 2002 | Canada | English | Clinical - non-dissociative disorders | 116 | N/A |  |  |  |  |  | 64.7% |
| DDS2 |  |  | Depersonalization Phenomena in a Sample Population of College Students | J.C. Dixon | 1963 | USA | English | Non-clinical / control | 127 | N/A |  |  |  |  |  | 45.7% |
| DEMO01 |  |  | Developing a new measure of dissociation: The Dissociative Experiences Measure, Oxford (DEMO) | Černis E et al. | 2018 | UK | English | Non-clinical / control | 691 | 32.3 +/- 10.3 |  |  |  | 691 | 32.3 +/- 10.3 | 88.6% |
| DEMO02 |  |  | Psychometric evaluation of the Hong Kong  Chinese version of the Dissociative Experiences Measure, Oxford (HKC-DEMO) | Ng A and Chan W. | 2023 | Hong Kong | Hong Kong Chinese | Non-clinical / control | 914 | 27.3 +/- 9.4 |  |  |  |  |  | 72% |
| DES01 |  |  | Development, Reliability, and Validity of a Dissociation Scale | Bernstein E and Putnam F | 1986 | USA | English | Clinical - dissociative disorders/experiences; Clinical - non-dissociative disorders; Non-clinical / control | 182 | N/A |  | 117 |  | 65 |  | N/A |
| DES02 |  |  | A Validation Study of the DES in the Netherlands. | Ensink B and Otterloo D. | 1989 | the Netherlands | Dutch | Clinical - dissociative disorders/experiences; Non-clinical / control | 100 | N/A |  | 20 |  | 80 |  | 69% (Clinical = 85%, Control = 65%) |
| DES03 |  |  | The Dissociative Experiences Scale: Further Replication and Validation | Frischholz E et al. | 1990 | USA | English | Clinical - dissociative disorders/experiences; Non-clinical / control | 321 |  |  | 62 |  | 259 |  |  |
| DES04 | PAS1990 |  | A Factor Analytic Study of Two Scales Measuring Dissociation | Fischer D and Elnitsky S. | 1990 | Canada | English | Non-clinical / control | 507 | N/A |  |  |  |  |  | 52% |
| DES05 |  |  | Construct Validity of the Dissociative Experiences Scale (DES): I The Relationship between the DES and Other Self-reported Measures of DES | Frischholz E et al. | 1991 | USA | English | Non-clinical / control | 311 | N/A |  |  |  |  |  |  |
| DES06 |  |  | Dissociative Experiences in the General Population: a factor analysis | Ross C et al. | 1991 | Canada | English | Non-clinical / control | 1055 | 43.2 +/- 16.7 |  |  |  |  |  | 58.30% |
| DES07 |  |  | Detection of Dissociative Disorders in Psychiatric Patients by a Screening Instrument and a Structured Diagnostic Interview | Steinberg M et al. | 1991 | USA | English | Clinical - dissociative disorders/experiences; Clinical - non-dissociative disorders; Non-clinical / control | 45 | 39.9 |  |  |  |  |  | 71.10% |
| DES08 |  |  | Dissociative experiences in a college age population: a factor analystic | Ray W et al. | 1992 | USA | English | Non-clinical / control | 264 | N/A |  |  |  |  |  | N/A |
| DES09 |  |  | The Validation of the Dissociative Experiences Scale Against the Criterion of the SCID-D, using Receiver Operating Characteristics (ROC) Analysis | Draijer N and Boon S. | 1993 | The Netherlands | Dutch | Clinical – dissociative disorder / Non-clinical - healthy | 79 |  |  | 43 | 32.9 +/- 8.3 | 36 | 36.3 +/- 10.2 |  |
| DES10 |  |  | Validity of the Dissociative Experiences Scale in Screening for Multiple Personality Disorder: A Multicenter Study | Carlson E et al. | 1993 | USA | English | Clinical - dissociative disorders/experiences; Clinical - non-dissociative disorders | 1051 | 34.8 +/- 11.6 (of 912 participants available for age data) |  |  |  |  |  | 63% (of 887 participants available for sex data) |
| DES11  (DES II) |  |  | Convergent validity of the New Form of the DES | Ellason J et al. | 1994 | USA | English | Clinical - dissociative disorders/experiences; Clinical - non-dissociative disorders; Non-clinical / control | 178 | Approximately 50 |  | IPD disso - 87; IPD chem dep - 26 |  | 65 College students |  |  |
| DES12 |  |  | Assessing Dissociative Symptoms in Eating Disordered Patients: Construct Validation of Two SeIf- report Measures | Gleaves D and Eberenz K. | 1994 | USA | English | Clinical - non-dissociative disorders | 125 | 26.3 +/- 8.6 |  | 125 | 26.3 +/- 8.6 |  |  | 100 |
| DES13 |  |  | A Principal Components Analysis of the Dissociative Experiences Scale in a Substance Abuse Population | Dunn G et al. | 1994 | USA | English | Clinical - non-dissociative disorders (substance abuse) | 493 (patients with substance abuse) | 44.9 +/- 11.0 |  |  |  |  |  | 0% |
| DES14 |  |  | Psychometric Properties of the Dissociative Experiences Scale | Dubester K and Braun B. | 1995 | USA | English | Clinical - dissociative disorders/experiences; Clinical - non-dissociative disorders | 78 | 35.5 |  |  |  |  |  | 88.50% |
| DES15 |  |  | A factor analysis of the Dissociative Experiences Scale (DES) in dissociative identity disorder | Ross C et al. | 1995 | USA and Canada | English | Clinical - dissociative disorders/experiences | 274 | 33.2 +/- 8.5 |  |  |  |  |  | 89.40% |
| DES16 |  |  | The reliability and validity of The Turkish version of the Dissociative Experiences Scale | Yargic L et al. | 1995 | Turkey | Turkish | Clinical - dissociative disorders/experiences; Clinical - non-dissociative disorders; Non-clinical / control | 766 | N/A |  | 95 |  | 671 |  | N/A |
| DES17 | QED1995 |  | Measuring Clinical and Non-clinical dissociation: a comparison of the DES and QED | Gleaves D et al. | 1995 | USA | English | Clinical - dissociative disorders/experiences; Non-clinical / control | 200 | N/A |  | 15 (Disso) + 15 (Eating) | 38.6 +/- 6.3 (Disso)  N/A (Eating) | 170 | 21.3 +/- 3.9 | 64% (Clinical 100% Control 57.6%) |
| DES18 |  |  | Dimensionality of dissociation in subjects with PTSD | Amdur R and Liberzon I | 1996 | USA | English | Clinical - non-dissociative disorders | 129 | 45.2 +/- 7.2 |  |  |  |  |  | 0% |
| DES19 |  |  | Dissociative disorders in Japan: a pilot study with the dissociative experience scale and a semi-structured interview | Umesue M et al. | 1996 | Japan | Japanese | Clinical - dissociative disorders/experiences; Clinical - non-dissociative disorders; Non-clinical / control | 274 | N/A |  | 19 (Disso) + 16 (Schiz) |  | 40 (Normal adults) + 199 (Late adolescents) |  | N/A |
| DES20 |  |  | The Assessment of Dissociative Experiences in a Non-Clinical Population: Reliability, Validity, and Factor Structure of the Dissociative Experiences Scale | Holtgraves T and Stockdale. | 1997 | USA | English | Non-clinical / control | Experiment1 = 201 (students); Experiment2 = 195 (students/replicated) | N/A |  |  |  |  |  | Exp1 = 46.3% Exp 2 = 63.1% |
| DES21–  FDS |  |  | Adaptation and Psychometric Properties of the German Version of the Dissociative Experience Scale | Spitzer C et al. | 1998 | Germany | German | Clinical - dissociative disorders/experiences; Clinical - non-dissociative disorders; Clinical - other disorders / higher vulnerability; Non-clinical / control | 927 | 37 +/- 13.4 |  |  |  |  |  | 58 |
| DES22 |  |  | Validation of a French Version of the Dissociative Experiences Scale in a Rape-Victim Population | Derves-Bornoz J et al. | 1999 | France | French | Clinical - other disorders / higher vulnerability | 140 (Rape victims) | 23.6 +/- 9.6 |  |  |  |  |  | 90.70% |
| DES23 | DESC  1999 | DESVQ  1999 | Measuring dissociation: Comparison of alternative forms of the dissociative experiences scale | Wright D and Loftus E. | 1999 | USA | English | Non-clinical / control | Phase one: 75    Phase two (only for DES C): 260 | N/A |  |  |  |  |  | Phase one:  63%  Phase two:  N/A |
| DES24 –  CES |  |  | The Curious Experiences Survey, A Revised Version of the Dissociative Experiences Scale: Factor Structure, Reliability, and Relations to Demographic and Personality Variables | Goldberg L. | 1999 | USA | English | Non-clinical / control | 755 | N/A |  |  |  |  |  | 57.60% |
| DES25 | QED  2000 |  | Measuring Dissociative Experiences in a College  Population: A Study of Convergent and Discriminant Validity | Gleaves D et al. | 2000 | USA | English | Non-clinical / control | 220 | N/A |  |  |  |  |  | 67% |
| DES26 | DIS-Q  2001 |  | On the Dimensionalities of the Dissociative Experiences Scale (DES) and the Dissociation Questionnaire (DIS-Q) | Bernstein I et al. | 2001 | USA | English | Clinical - dissociative disorders/experiences; Non-clinical / control | DES = 1329 ; DIS-Q = 405 | DES N/A ; DIS-Q = 21.6 +/- 5 |  | DES 274 ; DIS-Q N/A | DES N/A | DES 1055 ; DIS-Q 405 | DES N/A | DES N/A;  DIS-Q = 66.7% |
| DES27 |  |  | Confirmatory Factor Analysis of Single- and Multiple-Factor Competing Models of the Dissociative Experiences Scale in a Nonclinical Sample | Stockdale G et al. | 2002 | USA | English | Non-clinical / control | Study 1: 971  Study 2: 400 | Study1: 19.2 +/- 2.5  Study2: N/A |  |  |  |  |  | Study1: 69%    Study2: 56% |
| DES28 | DES-T  2002 |  | The Dissociative Experiences scale-II: descriptive statistics factor analysis, and frequency of experiences | Zingrone N and Alvarado C. | 2002 | USA | English | Non-clinical / control | 308 | 23 +/- 8.3 |  |  |  |  |  | 60% |
| DES29 | DES-T  2003 |  | Relationship of Purported Measures of Pathological and Nonpathological Dissociation to Self-Reported Psychological Distress and Fantasy Immersion | Levin R and Spei E. | 2003 | USA | English | Non-clinical / control | 376 | 30.7 +/- 12 |  |  |  |  |  | 59.30% |
| DES30 | DES-T  2003 |  | Testing the dissociative taxon | Modestin J and Erni T. | 2003 | Switzerland | German | Clinical - non-dissociative disorders; Non-clinical / control | 480 | 34.5 |  | 207 | 48 +/- 12 | 276 | 24 +/- 3 | 49.6%  (Clinical 48% Non Clin 50%) |
| DES31 | DES-T  2003 | DES-NP2003 | The Finnish version of the Dissociative Experiences Scale-II (DES-II) and psychiatric distress | Lipsanen T et al. | 2003 | Finland | Finnish | Non-clinical / control | 924 | 40.1 +/- 12.6 |  |  |  |  |  | 60.10% |
| DES32 |  |  | Reliability and validity of a Swedish version of the Dissociative Experiences Scale (DES-II) | Körlin D et al. | 2007 | Sweden | Swedish | Clinical - other disorders / higher vulnerability; Non-clinical / control | 523 | 42 |  | 181 | 38.6 | 342 | 43.8 | 63.70% |
| DES33 |  |  | Investigating Dissociation Online: Validation of a Web-Based Version of the Dissociative Experiences Scale | Collins F and Jones K. | 2008 | Australia, USA, UK | English | Non-clinical / control | Pen and Paper: 42  Online: 293 | Pen and Paper: 27.7 +/- 8.2  Online: 32.7 +/- 9.9 |  |  |  |  |  | Pen and  Paper:  61.9%  Online:  80.9% |
| DES34 |  |  | Factor Structure and Correlates of the Dissociative Experiences Scale in a Large Offender Sample | Ruiz M et al. | 2008 | USA | English | Clinical - other disorders / higher vulnerability | 1515 | 30.5 +/- 6.6 |  |  |  |  |  | 17.50% |
| DES35 |  |  | Validation of the Hebrew Version of the Dissociative Experiences Scale (H-DES) in Israel | Somer E et al. | 2008 | Israel | Hebrew | Clinical - dissociative disorders/experiences; Clinical - non-dissociative disorders; Non-clinical / control | 630 | 28 |  | 340 | 31.9 +/- 10.8 | 290 | 23.4 +/- 7.9 | 64.9% (Clinical 60.9% Control 69.7%) |
| DES36 |  |  | Portuguese Validation of the Dissociative Experiences Scale | Santo H and Abreu J. | 2009 | Portugal | Portuguese | Clinical - dissociative disorders/experiences; Clinical - non-dissociative disorders; Non-clinical / control | 570 | 32.5 +/- 12.5 |  | Pts with disso symptoms = 113; Pts with various psych = 233 | Pts with dissociative symptoms = 30.8 +/- 12.3; Pts with various psychiatric disorders = 32.7 +/- 12.8 | Normal 224 | Normal = 33.0 +/- 12.2 | Total = 59.8%; Pts with dissociative symptoms = 71.0%; Pts with various psychiatric disorders =. 66.0%; Normal=60.0% |
| DES37 | SDQ-20  2013 | MID  2013 | Screening for Dissociative Disorders in Psychiatric Out- and Day Care-Patients | Mueller-Pfeiffer et al. | 2013 | Switzerland | German | Clinical - dissociative disorders/experiences | 160 | Median 34.0 and IQR 20 |  |  |  |  |  | 76% |
| DES38 |  |  | Factorial structure and psychometric properties of the French adaptation of the Dissociative Experiences Scale (DES) in non-clinical participants | Larøi F et al. | 2013 | France | French | Non-clinical / control | Study 1: 188    Study 2: 210 | Study 1: 21.7 +/- 2.7  Study 2: 21.7 +/- 3.9 |  |  |  |  |  | Study 1: 54.8%  Study 2: 83.3% |
| DES39 |  |  | Factor Structure of the Dissociative Experiences Scale: An Examination Across Sexual Assault Status | Olsen S et al. | 2013 | USA | English | Non-clinical / control | Study EFA: 540 Study CFA: 435 | Study EFA: 21.2 +/- 5.9 Study CFA: 20.0 +/- 3.6 |  |  |  |  |  |  |
| DES40 |  |  | Construct Validity of the Dissociative Experiences Scale: II. Its Relationship to Hypnotizability | Frischholz E et al. | 2014 | USA | English | Non-clinical / control | 311 (undergraduates) | N/A |  |  |  |  |  | 62% |
| DES41 |  |  | Dissociative absorption: An empirically unique, clinically relevant, dissociative factor | Soffer-Dudek N et al. | 2015 | Israel | Hebrew | Non-clinical | 679 (undergraduates) | 23.5 +/- 1.8 |  |  |  |  |  | 81.8% |
| DES42 |  |  | Reliability and Validity of the Dissociative Experiences Scale Among South Korean Patients With Schizophrenia | Oh H et al. | 2015 | Korea | Korean | Clinical - non-dissociative disorders | 68 (Schizophrenia patients) | 34.5 |  |  |  |  |  | 62% |
| DES43 |  |  | Validity and Reliability of Persian Versions of Peritraumatic Distress Inventory (PDI) and Dissociative Experiences Scale (DES) | Kianpoor M et al. | 2016 | Iran | Persian | Clinical - other disorders / higher vulnerability | 43 | 26.88 +/- 8.33 |  |  |  |  |  | 0% |
| DES44 |  |  | Is the Dissociative Experiences Scale able to identify detachment and compartmentalization symptoms? Factor structure of the Dissociative Experiences Scale in a large sample of psychiatric and nonpsychiatric subjects | Mazzotti E et al. | 2016 | Italy | Italian | Clinical - dissociative disorders/experiences; Clinical - non-dissociative disorders; Non-clinical / control | Psychiatric 780 (Dissociative 30) and Nonpsychiatric 2303 (Undergraduates and OPD Gyne-Dermato-Cancer) | Psychiatric 39.2 +/- 13.9 and Nonpsychiatric 30.3 +/- 14.2 |  | 780 | 39.2 +/- 13.9 | 2303 | 30.3 +/- 14.2 | 76.3 % (Psychiatric 71.5% and Nonpsychiatric 77.9%) |
| DES45 |  |  | Using Online Methods to Develop and Examine the Hong Kong Chinese Translation of the Dissociative Experiences Scale | Chan C et al. | 2017 | Hong Kong | Hon Kong Chinese | Non-clinical / control | 108 | 26.4 |  |  |  | 108 |  | 80% |
| DES46 | DESC  2017 |  | Psychometric Comparison of Dissociative Experiences Scales II and C: A Weak Trauma-Dissociation Link | Patihis L and Lynn S. | 2017 | USA | English | Non-clinical / control | 602 | 20.6 +/- 3.4 |  |  |  |  |  | 84.20% |
| DES47 |  |  | Validity and reliability of a Persian version of the Dissociative Experiences Scale II (DES-II) on Iranian patients diagnosed with schizophrenia and mood disorders | Ghaffarinejad A et al. | 2019 | Iran | Persian | Clinical - non-dissociative disorders; Non-clinical / control | 370 | 30.9 |  | 50 MDD and 50 Schiz | 32.0 +/- 7.9 MDD and 32.2 +/- 7.6 Schizophrenia | 270 | 30.4 +/- 7.3 | 57.5% (Healthy 68.2%, MDD 30%, Schizophrenia 28%) |
| DES48 |  |  | Improving the psychometric properties of the dissociative experiences scale (DES-II): a Rasch validation study | Saggino A et al. | 2020 | Italy | Italian | Non-clinical / control  (inmates and communities) | 320 |  |  | 122 (Incarcerated) | 40.0 +/- 11.8 | 198 (community) | 32.5 +/- 10.3 | 26.6% (Clinical 2% Nonclinical 41.4%) |
| DES49 |  |  | Confirmatory factor analyses of the dissociative experiences scale in schizophrenia: Results from two psychiatric samples in South Korea | Jeong H et al. | 2021 | Korean | Korea | Clinical - non-dissociative disorders | 552 Patients with schizophrenia (300=National provincial hospital; 252=University-affiliated) | Schizophrenia = 44.9 +/- 10.4 |  |  |  |  |  | 50.2% (National provincial hospital=63.4%; University-affiliated=56.0%) |
| DES50 |  |  | Validating Dissociative Experience Scale (DES) in a Greek sample | Tzikos A et al. | 2021 | Greece | Greek | Clinical - dissociative disorders/experiences; Clinical - non-dissociative disorders; Non-clinical / control | 340 | 38.4 |  | 122 | 39 +/- 11 | 218 | 38 +/- 10 | 72.1% (Clinical 71.6% Control 74.8%) |
| DES51  and  extended  DES  (FDS) |  |  | Validation of the extended version of the Dissociative Experiences Scale (DES) in patients diagnosed with substance use disorders | Gidzgier P et al. | 2022 | Germany | German | Clinical - non-dissociative disorders | 459 | 36.7 +/- 11.2 |  |  |  |  |  | 40.30% |
| DES52 |  |  | The Dissociative Experiences Scale: An Empirical Evaluation of Long-Standing Concerns | Trujillo M et al. | 2022 | USA | English | Non-clinical / control | Study 1: 163  Study 2: 447 | N/A |  |  |  |  |  | Study 1: 72.2%    Study 2: 84.3% |
| DES53 | DESC  2023 | DESR2023 (original study not available) | Psychometrics of Three Dissociation Scales: Reliability and Validity Data on the DESR, DES-II, and DESC | Arzoumanian M et al. | 2023 | USA | English |  | 300 | Study 2: 24.8 +/- 6.7 |  |  |  |  |  | 69.70% |
| DES-T01 |  |  | Types of Dissociation and Dissociative Types: A Taxometric Analysis of Dissociative Experiences | Waller et al. | 1996 | USA | English | Clinical - dissociative disorders/experiences; Clinical - non-dissociative disorders; Non-clinical / control | 1574 | N/A |  | 1159 |  | 415 |  | N/A |
| DES-T02 |  |  | Dissociative Experiences Scale Taxon and Measurement of Dissociative Pathology: Does the Taxon Add to an Understanding of Dissociation and Its Associated Pathologies? | Leavitt F. | 1999 | USA | English | Clinical - dissociative disorders/experiences; Clinical - non-dissociative disorders | 221 | 37.5 +/- 8.9 |  | 143 Dissociative |  | 72 Nondissociative |  | 100% |
| DES-T03 |  |  | Prevalence, Reliability and Validity of Dissociative Disorders in an Inpatient Setting | Ross C et al. | 2002 | USA | English | Clinical - non-dissociative disorders | 201 | 39.4 +/- 12.0 |  |  |  |  |  |  |
| DES-T04 |  |  | Is There Really a Dissociative Taxon on the Dissociative Experiences Scale? | Merritt R and You S. | 2008 | USA | English | Non-clinical / control | 1237 | 19.3 +/- 1.8 |  |  |  |  |  | 46.90% |
| DES-T05 | SDI-52018 | SR-DDIS2018 | Psychometric properties of the pathological dissociation measures among Chinese – a pilot study using online methods | Fung H et al. | 2018 | Hong Kong | Hong Kong Chinese | Clinical - dissociative disorders/experiences; Non-clinical / control | 203 |  |  | 43 | 30.3 | 160 | 20.1 +/- 1.7 | 74.4% (Clinical 90.7% Control 70%) |
| DIS-Q01 |  |  | The dissociation questionnaire (DIS-Q): Development and characteristics of a new self-report questionnaire | Vanderlinden J et al. | 1993 | Belgium and the Netherlands | Dutch | Clinical - dissociative disorders/experiences; Clinical - non-dissociative disorders; Non-clinical / control | 378 (first phase in the control) and 261 (second phase in both dissociative disorders and other psychiatric disorders) | Not mentioned |  | 374 | Not mentioned | 217 | Not mentioned | 49.9% in the control; but not mentioned in the psychiatric group |
| DIS-Q02 |  |  | Evaluation of the Swedish Version of Dissociation  Questionnaire (DIS-Q), Dis-Q-Sweden, Among Adolescents | Nilsson D et al. | 2006 | Sweden | Swedish | Clinical - other disorders / higher vulnerability; Non-clinical / control | 474 | 15.2 |  | 74 | 16.0 +/- 1.8 | 400 | 15.1 +/- 1.9 | 53.6% (Clinical 86.5% Nonclinical 47.5%) |
| DIS-Q03 | DES2011 |  | Assessment of Dissociation Symptoms in Patients with  Mental Disorders by the Dissociation Questionnaire  (DIS-Q) | Matsui Y et al. | 2010 | Japan | Japanese | Clinical - dissociative disorders/experiences; Clinical - non-dissociative disorders; Non-clinical / control | 190 | 33.5 |  | 107 | 33.2 | 83 | 33.8 +/- 11.2 | 67.9% (Control 55.4% Clinical 77.6%) |
| DTS01 |  |  | Development and Psychometric Characteristics of  the Dissociation Tension Scale | Stiglmayr C et al. | 2010 | Germany | suspected Germany (but in the manuscript is English). | Clinical - non-dissociative disorders | 294 | 32.2 +/- 8.7 |  | 264 | 30.6 | 30 | 29.7 +/- 7.5 | 86.1 |
| DSS01 |  |  | Development of a Depersonalization Severity Scale | Simeon D et al. | 2001 | USA | English | Clinical - dissociative disorders/experiences | 63 | 34.4 +/- 10.4 |  | 63 | 34.4 +/- 10.4 |  |  | 52.40% |
| DSS01 |  |  | Development and Validation of the Dissociative Symptoms Scale | Carlson E et al. | 2018 | USA | English | Clinical - non-dissociative disorders; Non-clinical / control | 1592 | Age reported in groups of range of age |  | Veterans = 355; PTSD = 33; Trauma center = 139 |  | 1055 |  | 52.7 |
| DSS02 |  |  | Factor Structure and Clinical Correlates of The Dissociative Symptoms Scale (DSS) Korean Version Among Community Sample With Adverse Childhood Experiences | Jo et al. | 2023 | Korea | Korean | Non-clinical / control | 1304 | 40.0 +/- 10.3 |  |  |  |  |  | 66.70% |
| DSS03 |  |  | Psychometric properties of the dissociative symptoms scale (DSS) in Italian outpatients and community adults | Schimmenti A et al. | 2020 | Italy | Italian | Clinical - non-dissociative disorders; Non-clinical / control | 598 | 39.2 |  | 175 | 42 +/- 14.5 | 423 | 38 +/- 12.5 | (Clinical 50.1% Control 52.0%) |
| DSS-401 |  |  | Development and Psychometric Characteristics of the DSS-4 as a Short Instrument to Assess Dissociative Experience during Neuropsychological Experiments | Stiglmayr C et al. | 2009 | They used previously collected data. | German original but English translation provided in the manuscript | Clinical - non-dissociative disorders; Non-clinical / control | 142 | 27 |  | 102 |  | 40 |  | 100% |
| DSS-B01 |  |  | Development of a Brief Version of the Dissociative Symptoms Scale and the Reliability and Validity of DSS-B Scores in Diverse Clinical and Community Samples | Macia K et al. |  | USA | English | Clinical - other disorders / higher vulnerability | 478 | 40.2 +/- 14.0 |  |  |  |  |  | 34.70% |
| FDS01 |  |  | Primary and secondary depersonalisation disorder: a psychometric study | Lambert M et al. | 2001 | UK | English | Clinical - dissociative disorders/experiences; Clinical - non-dissociative disorders; Non-clinical / control | 125 | 36.2 |  | 97 | 38.4 | 28 | 28.5 | 51.2%  (Clinical 46.4% Control 67.9%) |
| JBS01 |  |  | Toward a clarification of the construct of depersonalization and its association with affective and cognitive dysfunction | Jacobs J and Bovasso G. | 1992 | USA | English | Non-clinical / control | 368 | 22 |  |  |  |  |  | 75% |
| GDS01 |  |  | Measuring Dissociation and Hypnotisability with African American College Students: A New Dissociation Scale - the General Dissociation Scale. | Sapp M and Hichcock K. | 2003 | USA | English | Non-clinical / control | 202 | 19.9 +/- 2.6 |  |  |  |  |  | 61.40% |
| MID01 |  |  | The Multidimensional Inventory of Dissociation (MID): A Comprehensive Measure of Pathological Dissociation | Dell P. | 2008 | USA | English | Clinical - dissociative disorders/experiences; Clinical - non-dissociative disorders; Non-clinical / control | Study 1: 204 (63 nonclinic + 67 nondisso + 74 disso)    Study 2: 614 (464 disso + 149 nondisso) | Study 1: 44.4  Study 2: 39.5 |  |  |  |  |  | Study 1: 77.0%  Study 2: 75.4% |
| MID02 |  |  | Development of the Hebrew-Multidimensional Inventory of Dissociation (H-MID): A Valid and Reliable Measure of Pathological Dissociation | Somer E and Dell P. | 2008 | Isarel | Hebrew | Non-clinical / control | 141 | 28.4 +/- 7.8 |  |  |  |  |  | 73.00% |
| MID03 |  |  | Three Dimensions of Dissociative Amnesia | Dell P. | 2013 | USA, Canada, Australia, Israel | English | Clinical - dissociative disorders/experiences / control | 2569 |  |  | 1331 |  | 1238 |  | 62.1% |
| MID04  (short MID) |  |  | Measuring Dissociative Symptoms and Experiences in an Australian College Sample Using a Short Version of the Multidimensional Inventory of Dissociation | Kate M et al. | 2021 | Australia | English | Non-clinical / control | 313 | 32.0 +/- 10.5 |  |  |  |  |  | 85.90% |
| MID05 |  |  | The Multidimensional Inventory of Dissociation (MID) in Depersonalization Disorder: General Findings with a Clinical Emphasis on Memory and Identity Disturbances | Simeon D and Knutelska M. | 2022 | USA | English | Clinical - dissociative disorders/experiences | 23 | 31.0 +/- 10.3 |  |  |  |  |  | 60.90% |
| MSDQ01 |  |  | A Novel Screening Tool for Assessing Child Abuse: The Medical Somatic Dissociation Questionnaire–MSDQ | Daphna-Tekoah S et al. | 2019 | Israel | Hebrew and Arabic | Non-clinical / control | 541 | 35 +/- 12 |  | 138 (child sexual abuse group) |  | 193 |  | 70.40% |
| NCDI01 |  |  | The North Carolina Dissociation Index: A Measure  of Dissociation Using Items From the MMPI-2 | Mann B. | 1995 | USA | English | Non-clinical / control | Study 1: 525 (students)    Study 2: 431 (students) | Study 1: 19.2 +/- 2.3    Study 2: 19.3 +/- 2.5 |  |  |  |  |  | Study 1: 65.5%    Study 2: 63.8% |
| PAS01 |  |  | The Perceptual Alteration Scale: A Scale Measuring Dissociation | Sanders S. | 1986 | USA | English | Non-clinical / control | 133 | N/A |  | 40 (Binge eatings) | 19.6 +/- 1.7 | 74 (Normal students) | 20.8 +/- 9.7 | N/A |
| PDEQ01 |  |  | Peritraumatic dissociation and posttraumatic stress in male Vietnam theater veterans | Marmar C et al. | 1994 | USA | English | Clinical - other disorders / higher vulnerability | 251 | 41 +/- 4.9 |  |  |  |  |  | 0% |
| PDEQ  (RAND  PDEQ)  02 |  |  | Development and validation of a modified version of the Peritraumatic Dissociative Experiences Questionnaire | Marshall G et al. | 2002 | USA | English | Clinical - other disorders / higher vulnerability | Study A: 284    Study B: 90 | Study A: 24.4 +/- 6.1  Study B: 24.3 +/- 5.6 |  |  |  |  |  | Study A: 7%    Study B: 100% |
| PDEQ03 |  |  | Validation of the Peritraumatic Dissociative Experiences Questionnaire self-report version in two samples of French-speaking individuals exposed to trauma | Birmes et al. | 2005 | France and Canada | French | Clinical - other disorders / higher vulnerability | 91 | 38.2 |  |  |  |  |  | 56.00% |
| PDEQ04 |  |  | The Latent Structure of the Peritraumatic Dissociative Experiences Questionnaire | Brooks et al. | 2009 | Australia | English | Clinical - other disorders / higher vulnerability | 247 | 39.1 +/- 13.5 |  |  |  |  |  | 22.30% |
| PDEQ05 |  |  | Validation of the Peritraumatic Dissociative Experiences Questionnaire and Peritraumatic Distress Inventory in school-aged victims of road traffic accidents | Bui E et al. | 2011 | France | English | Clinical - other disorders / higher vulnerability | 133 | 11.7 +/- 2.2 |  |  |  |  |  | 43.6% |
| PDEQ06 |  |  | Model Comparison and Structural Invariance of the Peritraumatic Dissociative Experiences Questionnaire in Portuguese Colonial War Veterans | Cavalho T et al. | 2017 | Portugal | Portuguese | Clinical - non-dissociative disorders; Clinical - other disorders / higher vulnerability | 300 + 87 | 63.8 (Sample 1 (63.6 +/- 6.0) - Sample 2: with PTSD 64.1 +/- 4 and without PTSD 64.6 +/- 4.8) |  | 42 | 64.1 +/- 4 | 300 + 45 | 63.6 +/- 6.0 and 64.6 +/- 4.8 | 0% |
| PDEQ07 |  |  | Validity, Reliability and Internal Consistency of Persian Versions of the Childhood Trauma Questionnaire, the Traumatic Exposure Severity Scale and the Peritraumatic Dissociative Experiences Questionnaire | Nobakht H et al. | 2021 | Iran | Persia | Clinical - other disorders / higher vulnerability | 230 | 25.3 |  |  |  |  |  | 50.90% |
| PDEQ08 |  |  | Psychometric Properties of the Peritraumatic Dissociative Experiences Questionnaire (PDEQ) in a Sample of Chilean Firefighters | Ramos N et al. | 2022 | Chile | Chilean Spanish | Clinical - other disorders / higher vulnerability | 736 | 32.5 +/- 12.0 |  |  |  |  |  | 14.40% |
| PDS(B)01 |  |  | Development and validation of the brief pathological dissociation scale PDS(B): Initial psychometrics | Kira I and Shuwiekh H | 2022 | Egypt | Arabic and English | Non-clinical / control | 228 | 21.3 +/- 3.5 |  |  |  |  |  | 86.80% |
| PDS01 |  |  | Initial development and validation of the Phillips Dissociation Scale (PDS) of the MMPI | Phillips D | 1994 | USA | English | Clinical - dissociative disorders/experiences; Clinical - non-dissociative disorders | 40 | 26 |  | 20 (Dissociative disorders patients) | 37 | 20 (General psychiatry patients) | 35 | 82.5 |
| PSDS01 |  |  | The Dissociative Subtype of PTSD Scale: Initial Evaluation in a National Sample of Trauma-Exposed Veterans | Wolf E et al. | 2017 | USA | English | Clinical - other disorders / higher vulnerability | 860 | 63 +/- 12.3 |  |  |  |  |  | 8.50% |
| PSDS02 |  |  | Psychometric Properties of the Dissociative Subtype of PTSD Scale: Replication and Extension in a Clinical Sample of Trauma-Exposed Veterans | Guetta R et al. | 2019 | USA | English | Clinical - non-dissociative disorders | 209 | 53.8 +/- 11.4 |  |  |  |  |  | 16.30% |
| PSDS03 |  |  | Turkish Adaptation of Dissociative Subtype of Post Traumatic Stress Disorder Scale | İçin et al. | 2023 | Turkey | Turkish | Non-clinical / control | 279 | 27 +/- 4.7 |  |  |  |  |  | 66.30% |
| QED01 |  |  | Measurement of Dissociation | Riley K. | 1992 | USA | English | Non-clinical / control | 1210 | N/A |  |  |  |  |  | 62.80% |
| SCL01 |  |  | Augmenting Hopkins SCL Scales to Measure Dissociative Symptoms: Data From Two Nonclinical Samples | Briere J and Runtz M | 1990 | USA | English | Non-clinical / control | Sample 1 = 278 and Sample 2 = 291 | Sample 1 = 19.8 and Sample 2 = 19.7 |  |  |  |  |  | 100 |
| SDI01 |  |  | The Development of the Somatoform Dissociation Index (SDI) | Leavitt F. | 2001 | USA | English | Clinical - dissociative disorders/experiences; Clinical - non-dissociative disorders | 200 | 36.7 |  | 100 (Disso) | 37.2 +/- 8.7 | 100 (Nondisso) | 36.1 +/- 9.3 | 100 |
| SDQ-2001 |  |  | The Development and Psychometric Characteristics of the Somatoform Dissociation Questionnaire (SDQ-20) | Nijenhuis E et al. | 1996 | The Netherlands | Dutch | Clinical - dissociative disorders/experiences; Clinical - non-dissociative disorders | 100 |  |  | 50 (dissociative patients) | 34.8 +/- 9.7 | 50 (nondissociative psychiatric patients) | 34.7 +/- 12.7 | Overall 83% (Disso 88% and Nondisso 78%) |
| SDQ-2002 |  |  | The Validity and Reliability of the German Version of the Somatoform Dissociation Questionnaire (SDQ-20) | Mueller-Pfeiffer C et al. | 2010 | Germany | German | Clinical - dissociative disorders/experiences; Clinical - non-dissociative disorders | 225 | 37.7 +/- 14.0 |  | 39 (disso) |  | 186 (nondisso) |  | 78.70% |
| SDQ-2003 | SDQ-51998 |  | Psychometric Characteristics of the Somatoform Dissociation Questionnaire: A Replication Study | Nijenhuis et al. | 1998 | The Netherlands | Dutch | Clinical - dissociative disorders/experiences; Clinical - non-dissociative disorders | 76 | 33.2 |  | 31 (disso) | 31.1 +/- 10.3 | 45 (nondisso) | 34.6 +/- 10.1 | 72.4% (Disso 90.3% Nondisso 60%) |
| SDQ-20-04 |  |  | Differentiating Dissociative Disorders from Other Diagnostic Groups Through Somatoform Dissociation in Turkey | Tsar et al. | 2008 | Turkey | Turkish | Clinical - dissociative disorders/experiences; Clinical - non-dissociative disorders; Non-clinical / control | 319 | 30.1 +/- 9.5 |  | 50 Disso + 94 Nondisso | Disso 24.5 +/- 4.8 and Nondisso 32.6 | 175 | 30.3 +/0 9.4 | 62.4% (Disso 80% + NonDisso 53.2% + Control 62.3%) |
| SDQ-20-05 |  |  | Posttraumatic Somatoform Dissociation In French Psychiatric Outpatients | El-Hage et al. | 2002 | France | French | Clinical - non-dissociative disorders | 140 | 40.4 +/- 14.1 |  |  |  |  |  | 56.40% |
| SDQ-20-06 |  |  | Dissociative disorders and other psychopathological groups: exploring the differences through the Somatoform Dissociation Questionnaire (SDQ-20) | Santo and Pio-Abreu. | 2006 | Portugal | Portuguese | Clinical - dissociative disorders/experiences; Clinical - non-dissociative disorders; Non-clinical / control | 231 | 30.7 |  | 110 (disso) | 30.0 +/- 12.3 | 121 (depression and anxiety) | 31.4 +/- 11.6 | 69.70% |
| SDQ-20-07 | SDQ-52015 |  | Somatoform dissociation among Swedish  adolescents and young adults: The psychometric properties of the Swedish versions of the SDQ-20 and SDQ-5 | Nilsson et al. | 2014 | Sweden | Swedish | Clinical - non-dissociative disorders; Non-clinical / control | 505 | 17 |  | 50 (Eating disorder)C | 19.9 +/- 3.4 | 455 (students) | 16.7 +/- 1.1 | 57.8% (Clinical 100% and Control 53.2%) |
| SDQ-2008 |  |  | Validity and reliability of the Spanish version of the Somatoform Dissociation Questionnaire (SDQ-20) | González-Vázquez et al. | 2017 | Spain | Spainish | Clinical - dissociative disorders/experiences; Clinical - non-dissociative disorders | 360 | 39.4 +/- 10.2 |  | 68 (Disso) |  | 292 (Nondisso) |  | 76.60% |
| SDQ-5-01 |  |  | The development of the somatoform dissociation questionnaire (SDQ-5) as a screening instrument for dissociative disorders | Nijenhuis et al. | 1997 | The Netherlands | Dutch | Clinical - dissociative disorders/experiences; Clinical - non-dissociative disorders | Phase 1: 100 (50 disso and 50 nondisso)    Phase 2: 33 for cross validation | Phase 1: 34.8 |  | 50 (disso) | 34.8 +/- 9.7 | 50 (Nondisso) | 34.7 +/- 12.7 | Phase 1: 83%    Phase 2: 97% |
| SODAS01 | CES2003 | QED2003 | The Development and Psychometric Evaluation of a New Measure of Dissociative Activities | Mayer J and Farmer R. | 2003 | N/A | English | Non-clinical / control | 77 | 21.0 +/- 5.6 |  |  |  |  |  | 74.00% |
| SODAS02 |  |  | Turkish adaptation of the scale of dissociative activities | Yilmaz F and Akcan G. | 2022 | Turkey | Turkish | Non-clinical / control | EFA 178 and CFA 216 | EFA 21.2 +/- 1.6 and CFA 22.3 +/- 3.5 |  |  |  |  |  | EFA 82.0% and CFA 75.9% |
| SR-DDIS01 |  |  | The self-report Dissociative Disorders Interview Schedule: A preliminary report | Ross C and Browning E. | 2017 | USA | English | Clinical - other disorders / higher vulnerability | 100 | 40.2 +/- 11.8 |  |  |  |  |  | 85.00% |
| SSD01 |  |  | Psychometric validation of the State Scale of Dissociation (SSD) | Krüger C and Mace C. | 2002 | UK | English | Clinical - dissociative disorders/experiences; Clinical - non-dissociative disorders; Non-clinical / control | 130 |  |  | 10 (Disso) + 19 (MDD) + 18 (Schizophrenia) + 20 (Alcohol) | 35.8 +/- 4.1 (Disso) + 44.2 +/- 7.6 (MDD) + 34.2 +/- 5.4 (Schiz) + 39.9 +/- 5.4 (Alcohol) | 63 (Undergrads) | 29.3 +/- 4.8 | N/A |
| SDS01 |  |  | Development of a Subclinical Dissociation Scale by Comparing Healthy Controls with Patients | Masuda et al. | 2020 | Japan | Japanese | Clinical - dissociative disorders/experiences; Clinical - non-dissociative disorders; Non-clinical / control | 464 | 20.3 |  | 23 (PTSD 11 and DID 12) | 28.8 +/- 10.0 | 441 | 19.9 +/- 1.2 | 78.7% (Clinical 78.3% - Control 78.7%) |
| VOD-Q01 |  |  | Development and Preliminary Psychometric Properties of an Instrument for the Measurement of Obsessional Dissociative Experiences: The Van Obsessional Dissociation Questionnaire (VOD-Q) | Boysan M et al. | 2018 | Turkey | Turkish | Clinical - non-dissociative disorders; Non-clinical / control | First = 554 and Second = 257 | First = 21.2 +/- 1.9 and Second = 21.8 +/- 2.3 |  | 30 | 27.3 +/- 6.9 | 227 | 21.1 +/- 3.3 | First = 47.3% and Second = 75.1% |
| WES01 |  |  | Towards a cognitive model and measure of dissociation | Kennedy F et al. | 2004 | UK | English | Clinical - non-dissociative disorders; Non-clinical / control | 160 |  |  | 80 | 34.6 +/- 10.2 | 80 | 22.8 +/- 6.4 | 81.30% |
| MDI01 |  |  | Multiscale Dissociation Inventory Professional Manual. | Briere J | 2002 | Textbook | English | Clinical - other disorders / higher vulnerability | 444 | N/A |  |  |  |  |  | N/A |
| MDI02 |  |  | Is dissociation a multidimensional construct? Data from the Multiscale Dissociation Inventory | Briere J | 2002 | USA | English | Clinical - non-dissociative disorders; Non-clinical / control | 1326 | 33.7 +/- 17.9 |  |  |  |  |  | 60.00% |
| ADI01 |  |  | Dissociation in the laboratory: a comparison of strategies | Leonard K et al. | 2000 | USA | English | Non-clinical | 78 | 19.05 +/- 1.58 |  |  |  |  |  | 53.84% |
| ADI02 | ADI-A01 |  | Fear response to dissociation challenge | Leonard K et al. | 2000 | USA | English | Non-clinical | 101 | 18.97 +/- 1.16 |  |  |  |  |  | 64.4% |
| SFQ01 |  |  | Dissociation and hallucinations in dyads engaged through interpersonal gazing | Caputo G. | 2015 | Italy | English | Non-clinical | 40 | 21.85 +/- 1.27 |  |  |  |  |  | 75.0% |
| SFQ02 |  |  | Strange-face illusions during eye-to-eye gazing in dyads: specific effects on derealization,depersonalization and dissociative identity | Caputo G. | 2019 | Italy | English | Non-clinical | 90 | 22 +/- 2.3 |  |  |  |  |  | 67.8% |
| SFQ03 |  |  | Mirror- and Eye-Gazing Perceptions in Advanced Psychometric Perspective: Preliminary Findings | Lange R et al. | 2022 | Italy | English | Non-clinical | 90 | 22 +/- 2.3 |  |  |  |  |  | 67.8% |
| SFQ-R01 |  |  | Strange-face-in-the-mirror illusions: specific effects on derealization, depersonalization, and dissociative identity | Caputo G. | 2023 | Italy | English | Non-clinical | 21 | 21.4 +/- 1.78 |  |  |  |  |  | 71.4% |

**Supplementary table 4 – COSMIN assessment results**

| **Study ID** | **TEST2** | **TEST3** | **Title** | **Authors** | **Publication years** | **Evidence of good psychometric properties** | | | **COSMIN methodological quality rating** | | | | | |
| --- | --- | --- | --- | --- | --- | --- | --- | --- | --- | --- | --- | --- | --- | --- |
|  |  |  |  |  |  | **Structural validity** | **Internal consistency** | **Reliability** | **Content**  **validity** | **Structural validity** | **Internal consistency** | **Cross-cultural**  **validity** | **Reliability** | **Measurement error** |
| A-DES01 |  |  | Development and Validation of a Measure of Adolescent Dissociation: The Adolescent Dissociative Experiences Scale | Armstrong J et al. | 1997 | indeterminate (?) | sufficient (+) | sufficient (+) | doubtful | inadequate | very good | N/A | adequate | inadequate |
| A-DES02 |  |  | Reliability and Validity of the Adolescent Dissociative Experiences Scale | Smith S and Carlson E. | 1996 | Not mentioned in the study | insufficient (-) | indeterminate (?) | N/A | inadequate | very good | N/A | adequate | adequate |
| A-DES03 |  |  | The Adolescent Dissociative Experiences Scale: Psychometric Properties and Difference in Scores Across Age Groups | Farrington A et al. | 2001 | insufficient (-) | indeterminate (?) | sufficient (+) | N/A | adequate | very good | N/A | adequate | adequate |
| A-DES04 |  |  | Reliability and validity of the Turkish version of the adolescent dissociative experiences scale | Zoroglu et al. | 2002 | Not mentioned in the study | sufficient (+) | sufficient (+) | N/A | inadequate | very good | adequate | adequate | adequate |
| A-DES05 | CDC2001 |  | Multimodal Assessment of Dissociation in Adolescents: Inpatients and Juvenile Sex Offenders | Friedrich W et al. | 2001 | Not mentioned in the study | Not mentioned in the study | indeterminate (?) | N/A | inadequate | N/A | N/A | inadequate | inadequate |
| A-DES06 |  |  | A validation study of the Adolescent Dissociative Experiences Scale | Seeley S et al. | 2004 | Not mentioned in the study | indeterminate (?) | indeterminate (?) | N/A | inadequate | very good | N/A | inadequate | adequate |
| A-DES07 |  |  | Dissociation Among Swedish Adolescents and the Connection to Trauma An Evaluation of the Swedish Version of Adolescent Dissociative Experience Scale | Nilsson D and Svedin C | 2006 | insufficient (-) | indeterminate (?) | sufficient (+) | N/A | adequate | very good | adequate | adequate | adequate |
| A-DES08 |  |  | The Korean Version of the Adolescent Dissociative Experience Scale: Psychometric Properties and the Connection to Trauma among Korean Adolescents | Shin J et al. | 2009 | sufficient (+) | indeterminate (?) | sufficient (+) | N/A | adequate | very good | adequate | adequate | adequate |
| A-DES09 |  |  | Psychometric Properties of the Adolescent Dissociative Experiences Scale (A-DES) in Japanese Adolescents from a Community Sample | Yoshizumi T et al. | 2010 | insufficient (-) | sufficient (+) | indeterminate (?) | N/A | very good | very good | adequate | inadequate | adequate |
| A-DES10 |  |  | Dissociation in non-clinical and clinical sample of Czech adolescents. Reliability and validity of the Czech version of the Adolescent Dissociative Experiences Scale | Soukup J et al. | 2010 | insufficient (-) | sufficient (+) | sufficient (+) | N/A | adequate | very good | adequate | adequate | adequate |
| A-DES11 |  |  | Psychometric properties of the Adolescent Dissociative Experiences Scale in a sample of Italian adolescents | Schimmenti A | 2015 | sufficient (+) | indeterminate (?) | sufficient (+) | N/A | very good | very good | N/A | adequate | adequate |
| A-DES12 |  |  | Validation of the Italian version of the dissociative experience scale for adolescents and young adults | Da Pasquale et al. | 2016 | sufficient (+) | insufficient (-) | sufficient (+) | N/A | very good | very good | adequate | adequate | adequate |
| A-DES13 |  |  | Validation of the Factor Structure of the Adolescent Dissociative Experiences Scale in a Sample of Trauma-Exposed Detained Youth | Kerig et al. | 2016 | sufficient (+) | Not mentioned in the study | indeterminate (?) | N/A | very good | inadequate | N/A | inadequate | adequate |
| A-DES14 |  |  | The psychometric properties of the adolescent  dissociative experiences scale (A-DES) in a sample  of Portuguese at-risk adolescents | Correia-Santos P et al. | 2022 | sufficient (+) | indeterminate (?) | sufficient (+) | N/A | very good | inadequate | adequate | adequate | adequate |
| A-DES15 |  |  | Dissociation: Factor Structure and the Role of Trauma Among Treatment-Seeking Adolescents | Kyte D et al. | 2023 | sufficient (+) | Not mentioned in the study | indeterminate (?) | N/A | very good | inadequate | N/A | inadequate | adequate |
| A-DES16  (shortended  A-DES) |  |  | The Psychometric Properties of a Shortened Version of the Spanish Adolescent Dissociative Experiences Scale | Martínez-Taboas A eet al. | 2004 | Not mentioned in the study | indeterminate (?) | sufficient (+) | N/A | inadequate | very good | adequate | adequate | adequate |
| A-DES17  (abbreviated  A-DES) |  |  | Factor Structure, Measurement Invariance, and Abbreviated Versions of The Adolescent Dissociative Experiences Scale (A-DES) | Lindfors K et al. | 2022 | insufficient (-) | insufficient (-) | indeterminate (?) | N/A | very good | very good | adequate | inadequate | adequate |
| ARAS01 |  |  | The Attentional Resource Allocation Scale (ARAS): Psychometric Properties of a Composite Measure for Dissociation and Absorption | Carleton R et al. | 2010 | sufficient (+) | insufficient (-) | indeterminate (?) | doubtful | inadequate | inadequate | N/A | inadequate | adequate |
| CADC01 |  |  | A Checklist for Screening Dissociative Disorders in Children and Adolescents | Reagor P et al. | 1992 | Not mentioned in the study | Not mentioned in the study | indeterminate (?) | adequate | inadequate | inadequate | N/A | inadequate | doubtful |
| CAD-P01 |  |  | Cognitive appraisals of dissociation in psychosis: a new brief measure | Černis E et al. | 2021 | sufficient (+) | indeterminate (?) | sufficient (+) | very good | very good | very good | N/A | doubtful | adequate |
| CADSS01 |  |  | Measurement of Dissociative States with the Clinician-Administered Dissociative States Scale (CADSS) | Bremner J et al. | 1998 | Not mentioned in the study | sufficient (+) | sufficient (+) | doubtful | inadequate | inadequate | N/A | doubtful | doubtful |
| CADSS02 |  |  | State and Trait Dissociation: Evaluating Convergent and Discriminant Validity | Condon L and Lynn S | 2014 | Not mentioned in the study | indeterminate (?) | indeterminate (?) | N/A | inadequate | inadequate | N/A | inadequate | adequate |
| CADSS03 |  |  | A simplified 6-Item clinician administered dissociative symptom scale (CADSS-6) for monitoring dissociative effects of sub-anesthetic ketamine infusions | Rodrigues N et al. | 2021 | Not mentioned in the study | Not mentioned in the study | indeterminate (?) | N/A | doubtful | inadequate | N/A | inadequate | doubtful |
| CADSS04 |  |  | The Clinician-Administered Dissociative States Scale (CADSS): Validation of the German Version | Mertens Y and Daniels J. | 2021 | sufficient (+) | sufficient (+) | indeterminate (?) | N/A | adequate | very good | very good | inadequate | adequate |
| CADSS05 |  |  | Inhibition, Attentional Control and Binding Abilities in Relation to Dissociative Symptoms Among PTSD Patients | Vancappel A et al. | 2023 | Not mentioned in the study | indeterminate (?) | indeterminate (?) | N/A | inadequate | very good | adequate | inadequate | adequate |
| CDC01 |  |  | Development, reliability, and validity of a child dissociation scale | Putnam et al. | 1993 | Not mentioned in the study | indeterminate (?) | insufficient (-) | doubtful | inadequate | very good | N/A | doubtful | doubtful |
| CDC02 |  |  | The Child Dissociative Checklist | Wherry J et al. | 1994 | Insufficient (-) | Not mentioned in the study | indeterminate (?) | N/A | inadequate | inadequate | N/A | inadequate | doubtful |
| CDC03 |  |  | Reliability and Validity of the Turkish Version of the Child Dissociative Checklist | Zoroglu S et al. | 2002 | Not mentioned in the study | indeterminate (?) | insufficient (-) | N/A | inadequate | very good | adequate | doubtful | doubtful |
| CDC04 |  |  | Dissociative Experiences in Children with Abuse Histories: A Replication in Puerto Rico | Reyes-Pérez C et al. | 2005 | Not mentioned in the study | indeterminate (?) | indeterminate (?) | N/A | inadequate | very good | very good | inadequate | adequate |
| CDC05 |  |  | Pathological Dissociation as Measured by the Child Dissociative Checklist | Wherry J et al. | 2009 | insufficient (-) | sufficient (+) | indeterminate (?) | N/A | adequate | very good | N/A | inadequate | adequate |
| CDPS01 |  |  | Development and Validation of a Scale Measuring Childhood Dissociation in Adults: the Childhood Dissociative Predictor Scale | Branscomb L and Fagan J. | 1992 | Not mentioned in the study | Not mentioned in the study | indeterminate (?) | doubtful | inadequate | inadequate | N/A | inadequate | doubtful |
| CDS01 |  |  | The Cambridge Depersonalisation Scale: a new instrument for the measurement of depersonalisation | Sierra M and Berrios G | 2000 | Not mentioned in the study | sufficient (+) | sufficient (+) | doubtful | inadequate | very good | N/A | adequate | adequate |
| CDS02 |  |  | Unpacking the depersonalization syndrome: an exploratory factor analysis on the Cambridge Depersonalization Scale | Sierra M et al. | 2005 | sufficient (+) | Not mentioned in the study | indeterminate (?) | N/A | adequate | N/A | N/A | inadequate | doubtful |
| CDS03 |  |  | Reliability and validity of a Japanese version of the Cambridge depersonalization scale as a screening instrument for depersonalization disorder | Sugiura M et al. | 2009 | Not mentioned in the study | indeterminate (?) | sufficient (+) | N/A | inadequate | very good | adequate | adequate | adequate |
| CDS04 |  |  | Italian (cross cultural) adaptation and validation of the Cambridge Depersonalization Scale (CDS) | Migliorini V et al. | 2011 | Not mentioned in the study | indeterminate (?) | sufficient (+) | N/A | inadequate | very good | adequate | adequate | adequate |
| CDS05 | MDI2012 | DES2012 | Construct Validity of Three Depersonalization Measures in Trauma-Exposed College Students | Blevins C et al. | 2012 | Not mentioned in the study | indeterminate (?) | indeterminate (?) | N/A | very good | very good | N/A | inadequate | adequate |
| CDS06 |  |  | Factor Structure of the Cambridge Depersonalization Scale in Trauma-Exposed College Students | Blevins C et al. | 2013 | sufficient (+) | Not mentioned in the study | indeterminate (?) | N/A | inadequate | very good | N/A | inadequate | adequate |
| CDS07 |  |  | Psychometric Properties of the Cambridge Depersonalization Scale in Puerto Rico | Aponte-Soto M et al. | 2014 | sufficient (+) | indeterminate (?) | sufficient (+) | N/A | very good | very good | very good | adequate | adequate |
| CDS08 |  |  | Depersonalization: An exploratory factor analysis of the Italian version of  the Cambridge Depersonalization Scale | Fagioli F et al. | 2015 | sufficient (+) | indeterminate (?) | indeterminate (?) | N/A | adequate | inadequate | N/A | inadequate | doubtful |
| CDS09 |  |  | Reliability, validity, and psychometric properties of the Greek translation of the Cambridge Depersonalization Scale (CDS) | Kontoangelos K et al. | 2016 | sufficient (+) | insufficient (-) | sufficient (+) | N/A | adequate | very good | doubtful | adequate | adequate |
| CDS10 |  |  | Dissociative symptoms as measured by the Cambridge Depersonalization Scale in patients with a bipolar disorder | Tuineag M et al. | 2020 | sufficient (+) | sufficient (+) | indeterminate (?) | N/A | inadequate | very good | adequate | inadequate | adequate |
| CEFSA01 |  |  | A new perspective and assessment measure for common dissociative experiences: ‘Felt Sense of Anomaly’ | Černis E et al. | 2021 | sufficient (+) | sufficient (+) | sufficient (+) | doubtful | very good | very good | N/A | adequate | adequate |
| CPAS01 |  |  | The Children's Perceptual Alteration Scale (CPAS): A Measure of Children's Dissociation | Evers-Szostak M and Sanders S. | 1992 | Not mentioned in the study | Not mentioned in the study | sufficient (+) | doubtful | inadequate | inadequate | N/A | very good | inadequate |
| DAS01 |  |  | Principal component analysis of a measure of non-pathological dissociation: the dissociative ability scale | Fisher W et al. | 2013 | sufficient (+) | indeterminate (?) | indeterminate (?) | doubtful | adequate | very good | N/A | inadequate | adequate |
| DAS02 |  |  | Factor structure and reliability of the Spanish  version of the Dissociative Ability Scale | Pérez-Fabello M and Campos A. | 2017 | insufficient (-) | insufficient (-) | indeterminate (?) | N/A | adequate | very good | adequate | inadequate | adequate |
| DCI01 |  |  | The Detachment and Compartmentalization Inventory (DCI): An assessment tool for two potentially distinct forms of dissociation | Butler C et al. | 2019 | sufficient (+) | sufficient (+) | indeterminate (?) | doubtful | adequate | very good | N/A | inadequate | adequate |
| DCI02 |  |  | Spanish validation of the Detachment and  Compartmentalization Inventory (DCI) in a community and clinical sample. A new instrument for measuring dissociation | Perona-Garcelán et al. | 2020 | sufficient (+) | sufficient (+) | sufficient (+) | N/A | very good | very good | adequate | adequate | adequate |
| DDI01 |  |  | Instrument to assess Depersonalisation-derealisation in Panic Disorder | Cox B and Swinson R. | 2002 | Not mentioned in the study | indeterminate (?) | indeterminate (?) | doubtful | inadequate | very good | N/A | inadequate | adequate |
| DDS2 |  |  | Depersonalization Phenomena in a Sample Population of College Students | J.C. Dixon | 1963 | Not mentioned in the study | Not mentioned in the study | indeterminate (?) | doubtful | inadequate | inadequate | N/A | inadequate | inadequate |
| DEMO01 |  |  | Developing a new measure of dissociation: The Dissociative Experiences Measure, Oxford (DEMO) | Černis E et al. | 2018 | indeterminate (?) | sufficient (+) | indeterminate (?) | doubtful | adequate | very good | N/A | adequate | inadequate |
| DEMO02 |  |  | Psychometric evaluation of the Hong Kong  Chinese version of the Dissociative Experiences Measure, Oxford (HKC-DEMO) | Ng A and Chan W. | 2023 | indeterminate (?) | sufficient (+) | indeterminate (?) | N/A | adequate | very good | adequate | adequate | adequate |
| DES01 |  |  | Development, Reliability, and Validity of a Dissociation Scale | Bernstein E and Putnam F | 1986 | Not mentioned in the study | indeterminate (?) | sufficient (+) | doubtful | inadequate | doubtful | N/A | adequate | adequate |
| DES02 |  |  | A Validation Study of the DES in the Netherlands. | Ensink B and Otterloo D. | 1989 | Not mentioned in the study | indeterminate (?) | indeterminate (?) | N/A | inadequate | very good | N/A | inadequate | doubtful |
| DES03 |  |  | The Dissociative Experiences Scale: Further Replication and Validation | Frischholz E et al. | 1990 | Not mentioned in the study | indeterminate (?) | sufficient (+) | N/A | inadequate | very good | N/A | doubtful | doubtful |
| DES04 | PAS1990 |  | A Factor Analytic Study of Two Scales Measuring Dissociation | Fischer D and Elnitsky S. | 1990 | indeterminate (?) | indeterminate (?) | indeterminate (?) | N/A | adequate | very good | adequate | inadequate | adequate |
| DES05 |  |  | Construct Validity of the Dissociative Experiences Scale (DES): I The Relationship between the DES and Other Self-reported Measures of DES | Frischholz E et al. | 1991 | Not mentioned in the study | Not mentioned in the study | indeterminate (?) | N/A | inadequate | inadequate | N/A | inadequate | adequate |
| DES06 |  |  | Dissociative Experiences in the General Population: a factor analysis | Ross C et al. | 1991 | insufficient (-) | Not mentioned in the study | indeterminate (?) | N/A | adequate | inadequate | doubtful | inadequate | adequate |
| DES07 |  |  | Detection of Dissociative Disorders in Psychiatric Patients by a Screening Instrument and a Structured Diagnostic Interview | Steinberg M et al. | 1991 | Not mentioned in the study | Not mentioned in the study | indeterminate (?) | N/A | inadequate | inadequate | N/A | inadequate | adequate |
| DES08 |  |  | Dissociative experiences in a college age population: a factor analystic | Ray W et al. | 1992 | indeterminate (?) | Not mentioned in the study | indeterminate (?) | N/A | adequate | inadequate | N/A | inadequate | adequate |
| DES09 |  |  | The Validation of the Dissociative Experiences Scale Against the Criterion of the SCID-D, using Receiver Operating Characteristics (ROC) Analysis | Draijer N and Boon S. | 1993 | Not mentioned in the study | sufficient (+) | indeterminate (?) | N/A | inadequate | very good | very good | inadequate | adequate |
| DES10 |  |  | Validity of the Dissociative Experiences Scale in Screening for Multiple Personality Disorder: A Multicenter Study | Carlson E et al. | 1993 | Not mentioned in the study | Not mentioned in the study | indeterminate (?) | N/A | inadequate | N/A | N/A | inadequate | doubtful |
| DES11  (DES II) |  |  | Convergent validity of the New Form of the DES | Ellason J et al. | 1994 | Not mentioned in the study | Not mentioned in the study | indeterminate (?) | N/A | inadequate | inadequate | N/A | inadequate | doubtful |
| DES12 |  |  | Assessing Dissociative Symptoms in Eating Disordered Patients: Construct Validation of Two SeIf- report Measures | Gleaves D and Eberenz K. | 1994 | sufficient (+) | Not mentioned in the study | indeterminate (?) | N/A | adequate | inadequate | N/A | inadequate | adequate |
| DES13 |  |  | A Principal Components Analysis of the Dissociative Experiences Scale in a Substance Abuse Population | Dunn G et al. | 1994 | sufficient (+) | Not mentioned in the study | indeterminate (?) | N/A | adequate | inadequate | N/A | inadequate | doubtful |
| DES14 |  |  | Psychometric Properties of the Dissociative Experiences Scale | Dubester K and Braun B. | 1995 | Not mentioned in the study | sufficient (+) | sufficient (+) | N/A | inadequate | very good | N/A | doubtful | doubtful |
| DES15 |  |  | A factor analysis of the Dissociative Experiences Scale (DES) in dissociative identity disorder | Ross C et al. | 1995 | sufficient (+) | Not mentioned in the study | indeterminate (?) | N/A | adequate | inadequate | N/A | inadequate | adequate |
| DES16 |  |  | The reliability and validity of The Turkish version of the Dissociative Experiences Scale | Yargic L et al. | 1995 | Not mentioned in the study | indeterminate (?) | sufficient (+) | N/A | inadequate | very good | very good | adequate | adequate |
| DES17 | QED1995 |  | Measuring Clinical and Non-clinical dissociation: a comparison of the DES and QED | Gleaves D et al. | 1995 | Not mentioned in the study | indeterminate (?) | indeterminate (?) | N/A | inadequate | very good | N/A | inadequate | adequate |
| DES18 |  |  | Dimensionality of dissociation in subjects with PTSD | Amdur R and Liberzon I | 1996 | sufficient (+) | insufficient (-) | indeterminate (?) | N/A | inadequate | very good | N/A | inadequate | adequate |
| DES19 |  |  | Dissociative disorders in Japan: a pilot study with the dissociative experience scale and a semi-structured interview | Umesue M et al. | 1996 | Not mentioned in the study | indeterminate (?) | sufficient (+) | N/A | inadequate | very good | very good | adequate | adequate |
| DES20 |  |  | The Assessment of Dissociative Experiences in a Non-Clinical Population: Reliability, Validity, and Factor Structure of the Dissociative Experiences Scale | Holtgraves T and Stockdale. | 1997 | insufficient (-) | Not mentioned in the study | indeterminate (?) | N/A | adequate | inadequate | N/A | inadequate | very good |
| DES21–  FDS |  |  | Adaptation and Psychometric Properties of the German Version of the Dissociative Experience Scale | Spitzer C et al. | 1998 | Not mentioned in the study | indeterminate (?) | sufficient (+) | doubtful --- categorised in des | inadequate | very good | doubtful | adequate | adequate |
| DES22 |  |  | Validation of a French Version of the Dissociative Experiences Scale in a Rape-Victim Population | Derves-Bornoz J et al. | 1999 | sufficient (+) | sufficient (+) | indeterminate (?) | N/A | adequate | very good | doubtful | inadequate | adequate |
| DES23 | DESC  1999 | DESVQ  1999 | Measuring dissociation: Comparison of alternative forms of the dissociative experiences scale | Wright D and Loftus E. | 1999 | insufficient (-) | sufficient (+) | indeterminate (?) | N/A | adequate | very good | N/A | inadequate | adequate |
| DES24 –  CES |  |  | The Curious Experiences Survey, A Revised Version of the Dissociative Experiences Scale: Factor Structure, Reliability, and Relations to Demographic and Personality Variables | Goldberg L. | 1999 | indeterminate (?) | indeterminate (?) | indeterminate (?) | doubtful -- categorised in des | adequate | doubtful | N/A | inadequate | adequate |
| DES25 | QED  2000 |  | Measuring Dissociative Experiences in a College  Population: A Study of Convergent and Discriminant Validity | Gleaves D et al. | 2000 | indeterminate (?) | indeterminate (?) | indeterminate (?) | N/A | inadequate | very good | N/A | inadequate | adequate |
| DES26 | DIS-Q  2001 |  | On the Dimensionalities of the Dissociative Experiences Scale (DES) and the Dissociation Questionnaire (DIS-Q) | Bernstein I et al. | 2001 | insufficient (-) | sufficient (+) | indeterminate (?) | N/A | N/A | N/A | N/A | inadequate | doubtful |
| DES27 |  |  | Confirmatory Factor Analysis of Single- and Multiple-Factor Competing Models of the Dissociative Experiences Scale in a Nonclinical Sample | Stockdale G et al. | 2002 | insufficient (-) | sufficient (+) | indeterminate (?) | N/A | very good | very good | N/A | inadequate | adequate |
| DES28 | DES-T  2002 |  | The Dissociative Experiences scale-II: descriptive statistics factor analysis, and frequency of experiences | Zingrone N and Alvarado C. | 2002 | sufficient (+) | indeterminate (?) | indeterminate (?) | N/A | adequate | very good | N/A | inadequate | adequate |
| DES29 | DES-T  2003 |  | Relationship of Purported Measures of Pathological and Nonpathological Dissociation to Self-Reported Psychological Distress and Fantasy Immersion | Levin R and Spei E. | 2003 | Not mentioned in the study | Not mentioned in the study | indeterminate (?) | N/A | inadequate | inadequate | adequate | inadequate | adequate |
| DES30 | DES-T  2003 |  | Testing the dissociative taxon | Modestin J and Erni T. | 2003 | Not mentioned in the study | indeterminate (?) | indeterminate (?) | N/A | inadequate | very good | adequate | inadequate | adequate |
| DES31 | DES-T  2003 | DES-NP2003 | The Finnish version of the Dissociative Experiences Scale-II (DES-II) and psychiatric distress | Lipsanen T et al. | 2003 | indeterminate (?) | indeterminate (?) | indeterminate (?) | N/A | adequate | very good | adequate | inadequate | adequate |
| DES32 |  |  | Reliability and validity of a Swedish version of the Dissociative Experiences Scale (DES-II) | Körlin D et al. | 2007 | sufficient (+) | insufficient (-) | indeterminate (?) | N/A | adequate | very good | adequate | inadequate | adequate |
| DES33 |  |  | Investigating Dissociation Online: Validation of a Web-Based Version of the Dissociative Experiences Scale | Collins F and Jones K. | 2008 | Not mentioned in the study | indeterminate (?) | indeterminate (?) | N/A | inadequate | very good | N/A | inadequate | adequate |
| DES34 |  |  | Factor Structure and Correlates of the Dissociative Experiences Scale in a Large Offender Sample | Ruiz M et al. | 2008 | sufficient (+) | indeterminate (?) | indeterminate (?) | N/A | very good | very good | doubtful | inadequate | adequate |
| DES35 |  |  | Validation of the Hebrew Version of the Dissociative Experiences Scale (H-DES) in Israel | Somer E et al. | 2008 | Not mentioned in the study | indeterminate (?) | sufficient (+) | N/A | inadequate | very good | adequate | adequate | adequate |
| DES36 |  |  | Portuguese Validation of the Dissociative Experiences Scale | Santo H and Abreu J. | 2009 | sufficient (+) | sufficient (+) | sufficient (+) | N/A | adequate | very good | very good | adequate | adequate |
| DES37 | SDQ-20  2013 | MID  2013 | Screening for Dissociative Disorders in Psychiatric Out- and Day Care-Patients | Mueller-Pfeiffer et al. | 2013 | Not mentioned in the study | indeterminate (?) | indeterminate (?) | N/A | inadequate | very good | adequate | inadequate | adequate |
| DES38 |  |  | Factorial structure and psychometric properties of the French adaptation of the Dissociative Experiences Scale (DES) in non-clinical participants | Larøi F et al. | 2013 | sufficient (+) | sufficient (+) | indeterminate (?) | N/A | very good | very good | adequate | inadequate | adequate |
| DES39 |  |  | Factor Structure of the Dissociative Experiences Scale: An Examination Across Sexual Assault Status | Olsen S et al. | 2013 | sufficient (+) | Not mentioned in the study | indeterminate (?) | N/A | very good | inadequate | N/A | inadequate | adequate |
| DES40 |  |  | Construct Validity of the Dissociative Experiences Scale: II. Its Relationship to Hypnotizability | Frischholz E et al. | 2014 | Not mentioned in the study | Not mentioned in the study | indeterminate (?) | N/A | inadequate | inadequate | N/A | inadequate | very good |
| DES41 |  |  | Dissociative absorption: An empirically unique, clinically relevant, dissociative factor | Soffer-Dudek N et al. | 2015 | insufficient (-) | insufficient (-) | indeterminate (?) | N/A | very good | very good | doubtful | inadequate | doubtful |
| DES42 |  |  | Reliability and Validity of the Dissociative Experiences Scale Among South Korean Patients With Schizophrenia | Oh H et al. | 2015 | Not mentioned in the study | sufficient (+) | indeterminate (?) | N/A | inadequate | very good | adequate | doubtful | doubtful |
| DES43 |  |  | Validity and Reliability of Persian Versions of Peritraumatic Distress Inventory (PDI) and Dissociative Experiences Scale (DES) | Kianpoor M et al. | 2016 | Not mentioned in the study | indeterminate (?) | sufficient (+) | N/A | inadequate | very good | doubtful | adequate | adequate |
| DES44 |  |  | Is the Dissociative Experiences Scale able to identify detachment and compartmentalization symptoms? Factor structure of the Dissociative Experiences Scale in a large sample of psychiatric and nonpsychiatric subjects | Mazzotti E et al. | 2016 | sufficient (+) | sufficient (+) | indeterminate (?) | N/A | very good | inadequate | very good | inadequate | adequate |
| DES45 |  |  | Using Online Methods to Develop and Examine the Hong Kong Chinese Translation of the Dissociative Experiences Scale | Chan C et al. | 2017 | Not mentioned in the study | sufficient (+) | sufficient (+) | N/A | inadequate | very good | very good | very good | very good |
| DES46 | DESC  2017 |  | Psychometric Comparison of Dissociative Experiences Scales II and C: A Weak Trauma-Dissociation Link | Patihis L and Lynn S. | 2017 | indeterminate (?) | indeterminate (?) | sufficient (+) | N/A | inadequate | very good | N/A | adequate | adequate |
| DES47 |  |  | Validity and reliability of a Persian version of the Dissociative Experiences Scale II (DES-II) on Iranian patients diagnosed with schizophrenia and mood disorders | Ghaffarinejad A et al. | 2019 | Not mentioned in the study | sufficient (+) | sufficient (+) | N/A | inadequate | very good | adequate | adequate | adequate |
| DES48 |  |  | Improving the psychometric properties of the dissociative experiences scale (DES-II): a Rasch validation study | Saggino A et al. | 2020 | insufficient (-) | Not mentioned in the study | indeterminate (?) | N/A | very good | inadequate | doubtful | inadequate | adequate |
| DES49 |  |  | Confirmatory factor analyses of the dissociative experiences scale in schizophrenia: Results from two psychiatric samples in South Korea | Jeong H et al. | 2021 | sufficient (+) | Not mentioned in the study | indeterminate (?) | N/A | very good | inadequate | N/A | inadequate | doubtful |
| DES50 |  |  | Validating Dissociative Experience Scale (DES) in a Greek sample | Tzikos A et al. | 2021 | indeterminate (?) | indeterminate (?) | sufficient (+) | N/A | adequate | very good | very good | adequate | adequate |
| DES51  and  extended  DES  (FDS) |  |  | Validation of the extended version of the Dissociative Experiences Scale (DES) in patients diagnosed with substance use disorders | Gidzgier P et al. | 2022 | insufficient (-) | indeterminate (?) | sufficient (+) | N/A | adequate | very good | adequate | adequate | adequate |
| DES52 |  |  | The Dissociative Experiences Scale: An Empirical Evaluation of Long-Standing Concerns | Trujillo M et al. | 2022 | Not mentioned in the study | sufficient (+) | insufficient (-) | N/A | inadequate | very good | N/A | adequate | adequate |
| DES53 | DESC  2023 | DESR2023 (original study not available) | Psychometrics of Three Dissociation Scales: Reliability and Validity Data on the DESR, DES-II, and DESC | Arzoumanian M et al. | 2023 | Not mentioned in the study | sufficient (+) | sufficient (+) | N/A | inadequate | very good | adequate | adequate | adequate |
| DES-T01 |  |  | Types of Dissociation and Dissociative Types: A Taxometric Analysis of Dissociative Experiences | Waller et al. | 1996 | Not mentioned in the study | Not mentioned in the study | indeterminate (?) | adequate | inadequate | inadequate | N/A | inadequate | doubtful |
| DES-T02 |  |  | Dissociative Experiences Scale Taxon and Measurement of Dissociative Pathology: Does the Taxon Add to an Understanding of Dissociation and Its Associated Pathologies? | Leavitt F. | 1999 | Not mentioned in the study | Not mentioned in the study | indeterminate (?) | N/A | inadequate | inadequate | N/A | inadequate | doubtful |
| DES-T03 |  |  | Prevalence, Reliability and Validity of Dissociative Disorders in an Inpatient Setting | Ross C et al. | 2002 | Not mentioned in the study | Not mentioned in the study | indeterminate (?) | N/A | inadequate | inadequate | adequate | inadequate | adequate |
| DES-T04 |  |  | Is There Really a Dissociative Taxon on the Dissociative Experiences Scale? | Merritt R and You S. | 2008 | Not mentioned in the study | Not mentioned in the study | indeterminate (?) | N/A | inadequate | inadequate | adequate | inadequate | adequate |
| DES-T05 | SDI-52018 | SR-DDIS2018 | Psychometric properties of the pathological dissociation measures among Chinese – a pilot study using online methods | Fung H et al. | 2018 | Not mentioned in the study | indeterminate (?) | indeterminate (?) | N/A | inadequate | very good | doubtful | inadequate | adequate |
| DIS-Q01 |  |  | The dissociation questionnaire (DIS-Q): Development and characteristics of a new self-report questionnaire | Vanderlinden J et al. | 1993 | sufficient (+) | insufficient (-) | sufficient (+) | adequate | adequate | very good | N/A | very good | very good |
| DIS-Q02 |  |  | Evaluation of the Swedish Version of Dissociation Questionnaire (DIS-Q), Dis-Q-Sweden, Among Adolescents | Nilsson D et al. | 2006 | insufficient (-) | insufficient (-) | sufficient (+) | N/A | adequate | very good | adequate | very good | N/A |
| DIS-Q03 | DES2011 |  | Assessment of Dissociation Symptoms in Patients with  Mental Disorders by the Dissociation Questionnaire  (DIS-Q) | Matsui Y et al. | 2010 | Not mentioned in the study | indeterminate (?) | indeterminate (?) | N/A | inadequate | very good | adequate | inadequate | adequate |
| DTS01 |  |  | Development and Psychometric Characteristics of  the Dissociation Tension Scale | Stiglmayr C et al. | 2010 | sufficient (+) | indeterminate (?) | sufficient (+) | doubtful | adequate | very good | N/A | doubtful | doubtful |
| DSS01 |  |  | Development of a Depersonalization Severity Scale | Simeon D et al. | 2001 | Not mentioned in the study | insufficient (-) | sufficient (+) | doubtful | inadequate | very good | N/A | very good | very good |
| DSS01 |  |  | Development and Validation of the Dissociative Symptoms Scale | Carlson E et al. | 2018 | sufficient (+) | sufficient (+) | insufficient (-) | doubtful | very good | very good | N/A | adequate | adequate |
| DSS02 |  |  | Factor Structure and Clinical Correlates of The Dissociative Symptoms Scale (DSS) Korean Version Among Community Sample With Adverse Childhood Experiences | Jo et al. | 2023 | sufficient (+) | sufficient (+) | indeterminate (?) | N/A | very good | very good | doubtful | doubtful | doubtful |
| DSS03 |  |  | Psychometric properties of the dissociative symptoms scale (DSS) in Italian outpatients and community adults | Schimmenti A et al. | 2020 | sufficient (+) | insufficient (-) | sufficient (+) | N/A | very good | very good | very good | adequate | adequate |
| DSS-401 |  |  | Development and Psychometric Characteristics of the DSS-4 as a Short Instrument to Assess Dissociative Experience during Neuropsychological Experiments | Stiglmayr C et al. | 2009 | indeterminate (?) | sufficient (+) | sufficient (+) | doubtful | doubtful | very good | N/A | very good | doubtful |
| DSS-B01 |  |  | Development of a Brief Version of the Dissociative Symptoms Scale and the Reliability and Validity of DSS-B Scores in Diverse Clinical and Community Samples | Macia K et al. |  | sufficient (+) | indeterminate (?) | insufficient (-) | N/A | very good | very good | N/A | adequate | adequate |
| FDS01 |  |  | Primary and secondary depersonalisation disorder:  a psychometric study | Lambert M et al. | 2001 | Not mentioned in the study | Not mentioned in the study | indeterminate (?) | doubtful | inadequate | inadequate | N/A | inadequate | adequate |
| JBS01 |  |  | Toward a clarification of the construct of depersonalization and its association with affective and cognitive dysfunction | Jacobs J and Bovasso G. | 1992 | sufficient (+) | sufficient (+) | indeterminate (?) | doubtful | adequate | very good | N/A | inadequate | adequate |
| GDS01 |  |  | Measuring Dissociation and Hypnotisability with African American College Students: A New Dissociation Scale - the General Dissociation Scale. | Sapp M and Hichcock K. | 2003 | Not mentioned in the study | indeterminate (?) | indeterminate (?) | doubtful | inadequate | very good | N/A | inadequate | adequate |
| MID01 |  |  | The Multidimensional Inventory of Dissociation (MID): A Comprehensive Measure of Pathological Dissociation | Dell P. | 2008 | insufficient (-) | sufficient (+) | sufficient (+) | doubtful | very good | very good | N/A | adequate | adequate |
| MID02 |  |  | Development of the Hebrew-Multidimensional Inventory of Dissociation (H-MID): A Valid and Reliable Measure of Pathological Dissociation | Somer E and Dell P. | 2008 | Not mentioned in the study | insufficient (-) | indeterminate (?) | N/A | inadequate | very good | adequate | inadequate | adequate |
| MID03 |  |  | Three Dimensions of Dissociative Amnesia | Dell P. | 2013 | sufficient (+) | sufficient (+) | sufficient (+) | N/A | adequate | very good | N/A | adequate | adequate |
| MID04  (short MID) |  |  | Measuring Dissociative Symptoms and Experiences in an Australian College Sample Using a Short Version of the Multidimensional Inventory of Dissociation | Kate M et al. | 2021 | sufficient (+) | sufficient (+) | sufficient (+) | N/A | adequate | very good | very good | adequate | adequate |
| MID05 |  |  | The Multidimensional Inventory of Dissociation (MID) in Depersonalization Disorder: General Findings with a Clinical Emphasis on Memory and Identity Disturbances | Simeon D and Knutelska M. | 2022 | sufficient (+) | sufficient (+) | indeterminate (?) | N/A | adequate | very good | N/A | inadequate | adequate |
| MSDQ01 |  |  | A Novel Screening Tool for Assessing Child Abuse: The Medical Somatic Dissociation Questionnaire–MSDQ | Daphna-Tekoah S et al. | 2019 | insufficient (-) | indeterminate (?) | indeterminate (?) | doubtful | very good | doubtful | N/A | inadequate | adequate |
| NCDI01 |  |  | The North Carolina Dissociation Index: A Measure  of Dissociation Using Items From the MMPI-2 | Mann B. | 1995 | indeterminate (?) | indeterminate (?) | insufficient (-) | adequate | adequate | very good | N/A | adequate | adequate |
| PAS01 |  |  | The Perceptual Alteration Scale: A Scale Measuring Dissociation | Sanders S. | 1986 | insufficient (-) | indeterminate (?) | indeterminate (?) | doubtful | adequate | very good | N/A | inadequate | adequate |
| PDEQ01 |  |  | Peritraumatic dissociation and posttraumatic stress in male Vietnam theater veterans | Marmar C et al. | 1994 | insufficient (-) | sufficient (+) | indeterminate (?) | doubtful | adequate | very good | N/A | inadequate | adequate |
| PDEQ  (RAND  PDEQ)  02 |  |  | Development and validation of a modified version of the Peritraumatic Dissociative Experiences Questionnaire | Marshall G et al. | 2002 | insufficient (-) | sufficient (+) | indeterminate (?) | N/A | very good | very good | N/A | adequate | adequate |
| PDEQ03 |  |  | Validation of the Peritraumatic Dissociative Experiences Questionnaire self-report version in two samples of French-speaking individuals exposed to trauma | Birmes et al. | 2005 | sufficient (+) | indeterminate (?) | sufficient (+) | N/A | adequate | very good | very good | adequate | adequate |
| PDEQ04 |  |  | The Latent Structure of the Peritraumatic Dissociative Experiences Questionnaire | Brooks et al. | 2009 | sufficient (+) | indeterminate (?) | indeterminate (?) | N/A | very good | very good | very good | inadequate | adequate |
| PDEQ05 |  |  | Validation of the Peritraumatic Dissociative Experiences Questionnaire and Peritraumatic Distress Inventory in school-aged victims of road traffic accidents | Bui E et al. | 2011 | insufficient (-) | indeterminate (?) | insufficient (-) | N/A | adequate | very good | adequate | adequate | adequate |
| PDEQ06 |  |  | Model Comparison and Structural Invariance of the Peritraumatic Dissociative Experiences Questionnaire in Portuguese Colonial War Veterans | Cavalho T et al. | 2017 | sufficient (+) | sufficient (+) | sufficient (+) | N/A | very good | very good | very good | adequate | adequate |
| PDEQ07 |  |  | Validity, Reliability and Internal Consistency of Persian Versions of the Childhood Trauma Questionnaire, the Traumatic Exposure Severity Scale and the Peritraumatic Dissociative Experiences Questionnaire | Nobakht H et al. | 2021 | Not mentioned in the study | indeterminate (?) | insufficient (-) | N/A | inadequate | very good | doubtful | doubtful | doubtful |
| PDEQ08 |  |  | Psychometric Properties of the Peritraumatic Dissociative Experiences Questionnaire (PDEQ) in a Sample of Chilean Firefighters | Ramos N et al. | 2022 | sufficient (+) | sufficient (+) | indeterminate (?) | N/A | very good | very good | adequate | inadequate | adequate |
| PDS(B)01 |  |  | Development and validation of the brief pathological dissociation scale PDS(B): Initial psychometrics | Kira I and Shuwiekh H | 2022 | sufficient (+) | indeterminate (?) | insufficient (-) | very good | very good | very good | N/A | adequate | adequate |
| PDS01 |  |  | Initial development and validation of the Phillips Dissociation Scale (PDS) of the MMPI | Phillips D | 1994 | Not mentioned in the study | Not mentioned in the study | sufficient (+) | doubtful | inadequate | doubtful | N/A | adequate | adequate |
| PSDS01 |  |  | The Dissociative Subtype of PTSD Scale: Initial Evaluation in a National Sample of Trauma-Exposed Veterans | Wolf E et al. | 2017 | sufficient (+) | sufficient (+) | indeterminate (?) | adequate | very good | very good | N/A | inadequate | very good |
| PSDS02 |  |  | Psychometric Properties of the Dissociative Subtype of PTSD Scale: Replication and Extension in a Clinical Sample of Trauma-Exposed Veterans | Guetta R et al. | 2019 | sufficient (+) | sufficient (+) | indeterminate (?) | N/A | very good | very good | very good | inadequate | very good |
| PSDS03 |  |  | Turkish Adaptation of Dissociative Subtype of Post Traumatic Stress Disorder Scale | İçin et al. | 2023 | sufficient (+) | insufficient (-) | indeterminate (?) | N/A | very good | very good | doubtful | inadequate | doubtful |
| QED01 |  |  | Measurement of Dissociation | Riley K. | 1992 | Not mentioned in the study | indeterminate (?) | indeterminate (?) | doubtful | inadequate | very good | N/A | inadequate | adequate |
| SCL01 |  |  | Augmenting Hopkins SCL Scales to Measure Dissociative Symptoms: Data From Two Nonclinical Samples | Briere J and Runtz M | 1990 | Not mentioned in the study | indeterminate (?) | indeterminate (?) | inadequate | inadequate | doubtful | N/A | inadequate | adequate |
| SDI01 |  |  | The Development of the Somatoform Dissociation Index (SDI) | Leavitt F. | 2001 | Not mentioned in the study | indeterminate (?) | sufficient (+) | adequate | inadequate | very good | N/A | adequate | adequate |
| SDQ-2001 |  |  | The Development and Psychometric Characteristics of the Somatoform Dissociation Questionnaire (SDQ-20) | Nijenhuis E et al. | 1996 | Not mentioned in the study | Not mentioned in the study | indeterminate (?) | adequate | inadequate | inadequate | N/A | inadequate | adequate |
| SDQ-2002 |  |  | The Validity and Reliability of the German Version of the Somatoform Dissociation Questionnaire (SDQ-20) | Mueller-Pfeiffer C et al. | 2010 | Not mentioned in the study | indeterminate (?) | sufficient (+) | N/A | inadequate | very good | very good | adequate | adequate |
| SDQ-2003 | SDQ-51998 |  | Psychometric Characteristics of the Somatoform Dissociation Questionnaire: A Replication Study | Nijenhuis et al. | 1998 | indeterminate (?) | indeterminate (?) | indeterminate (?) | N/A | inadequate | inadequate | N/A | inadequate | adequate |
| SDQ-20-04 |  |  | Differentiating Dissociative Disorders from Other Diagnostic Groups Through Somatoform Dissociation in Turkey | Tsar et al. | 2008 | Not mentioned in the study | indeterminate (?) | sufficient (+) | N/A | inadequate | very good | very good | adequate | adequate |
| SDQ-20-05 |  |  | Posttraumatic Somatoform Dissociation In French Psychiatric Outpatients | El-Hage et al. | 2002 | sufficient (+) | insufficient (-) | indeterminate (?) | N/A | adequate | very good | adequate | inadequate | adequate |
| SDQ-20-06 |  |  | Dissociative disorders and other psychopathological groups: exploring the differences through the Somatoform Dissociation Questionnaire (SDQ-20) | Santo and Pio-Abreu. | 2006 | Not mentioned in the study | indeterminate (?) | indeterminate (?) | N/A | inadequate | very good | adequate | inadequate | adequate |
| SDQ-20-07 | SDQ-52015 |  | Somatoform dissociation among Swedish  adolescents and young adults: The psychometric properties of the Swedish versions of the SDQ-20 and SDQ-5 | Nilsson et al. | 2014 | Not mentioned in the study | insufficient (-) | indeterminate (?) | N/A | inadequate | very good | doubtful | adequate | adequate |
| SDQ-20-08 |  |  | Validity and reliability of the Spanish version of the Somatoform Dissociation Questionnaire (SDQ-20) | González-Vázquez et al. | 2017 | insufficient (-) | sufficient (+) | sufficient (+) | N/A | adequate | very good | adequate | adequate | adequate |
| SDQ-5-01 |  |  | The development of the somatoform dissociation questionnaire (SDQ-5) as a screening instrument for dissociative disorders | Nijenhuis et al. | 1997 | Not mentioned in the study | indeterminate (?) | indeterminate (?) | N/A | inadequate | very good | N/A | inadequate | adequate |
| SODAS01 | CES2003 | QED2003 | The Development and Psychometric Evaluation of a New Measure of Dissociative Activities | Mayer J and Farmer R. | 2003 | Not mentioned in the study | indeterminate (?) | sufficient (+) | doubtful | inadequate | very good | N/A | adequate | adequate |
| SODAS02 |  |  | Turkish adaptation of the scale of dissociative activities | Yilmaz F and Akcan G. | 2022 | sufficient (+) | indeterminate (?) | sufficient (+) | N/A | very good | very good | very good | adequate | adequate |
| SR-DDIS01 |  |  | The self-report Dissociative Disorders Interview Schedule: A preliminary report | Ross C and Browning E. | 2017 | Not mentioned in the study | Not mentioned in the study | indeterminate (?) | very good | inadequate | inadequate | N/A | inadequate | doubtful |
| SSD01 |  |  | Psychometric validation of the State Scale of Dissociation (SSD) | Krüger C and Mace C. | 2002 | sufficient (+) | sufficient (+) | sufficient (+) | doubtful | adequate | very good | N/A | adequate | adequate |
| SDS01 |  |  | Development of a Subclinical Dissociation Scale by Comparing Healthy Controls with Patients | Masuda et al. | 2020 | sufficient (+) | sufficient (+) | insufficient (-) | adequate | adequate | very good | N/A | adequate | adequate |
| VOD-Q01 |  |  | Development and Preliminary Psychometric Properties of an Instrument for the Measurement of Obsessional Dissociative Experiences: The Van Obsessional Dissociation Questionnaire (VOD-Q) | Boysan M et al. | 2018 | sufficient (+) | sufficient (+) | sufficient (+) | doubtful | very good | very good | N/A | very good | adequate |
| WES01 |  |  | Towards a cognitive model and measure of dissociation | Kennedy F et al. | 2004 | insufficient (-) | indeterminate (?) | indeterminate (?) | doubtful | very good | very good | N/A | inadequate | adequate |
| MDI01 |  |  | Multiscale Dissociation Inventory Professional Manual. | Briere J | 2002 | Not mentioned in the study | sufficient (+) | indeterminate (?) | doubtful | inadequate | very good | N/A | inadequate | inadequate |
| MDI02 |  |  | Is dissociation a multidimensional construct? Data from the Multiscale Dissociation Inventory | Briere J | 2002 | sufficient (+) | indeterminate (?) | indeterminate (?) | doubtful | inadequate | inadequate | N/A | inadequate | adequate |
| ADI01 |  |  | Dissociation in the laboratory: a comparison of strategies | Leonard K et al. | 1999 | Not mentioned in the study | Not mentioned in the study | Not mentioned in the study | doubtful | inadequate | N/A | N/A | Inadequate | Inadequate |
| ADI02 | ADI-A01 |  | Fear response to dissociation challenge | Leonard K et al. | 2000 | Not mentioned in the study | sufficient (+) | Not mentioned in the study | doubtful | inadequate | very good | N/A | Inadequate | Inadequate |
| SFQ01 |  |  | Dissociation and hallucinations in dyads engaged through interpersonal gazing | Caputo G. | 2015 | Not mentioned in the study | Not mentioned in the study | Not mentioned in the study | doubtful | inadequate | N/A | N/A | Inadequate | Inadequate |
| SFQ02 |  |  | Strange-face illusions during eye-to-eye gazing in dyads: specific effects on derealization, depersonalization and dissociative identity | Caputo G. | 2019 | sufficient (+) | sufficient (+) | Not mentioned in the study | N/A | adequate | very good | N/A | inadequate | inadequate |
| SFQ03 |  |  | Mirror- and Eye-Gazing Perceptions in Advanced PsychometricPerspective: Preliminary Findings | Lange R et al. | 2022 | sufficient (+) | Not mentioned in the study | Not mentioned in the study | N/A | very good | N/A | N/A | inadequate | inadequate |
| SFQ-R01 |  |  | Strange-face-in-the-mirror illusions: specific effects on derealization, depersonalization, and dissociative identity | Caputo G. | 2023 | Not mentioned in the study | sufficient (+) | Not mentioned in the study | doubtful | inadequate | very good | N/A | inadequate | inadequate |
